# Effect of Ga Doping on Microwave-Assisted Synthesized TiO_2_ Nanoparticles and their Photovoltaic Performance as DSSC Photoanodes

**DOI:** 10.1021/acsomega.6c06567

**Published:** 2026-10-27

**Authors:** J. Arriola-Oliva, E. Regalado-Pérez, U. Pal, J. Villanueva-Cab

**Affiliations:** † Instituto de Ciencias Químicas, 3972 Benemérita Universidad Autónoma de Puebla, San Pedro Zacachimalpa, 72960 Puebla, México; ‡ Instituto de Física “Ing. Luis Rivera Terrazas”, Universidad Autónoma de Puebla, 18 Sur y Av. San Claudio, C.U., 72570 Puebla, México

## Abstract

Undoped and Ga-doped (1–16% nominal) anatase TiO_2_ nanoparticles were synthesized using a rapid microwave-assisted method and employed as photoanodes in dye-sensitized solar cells (DSSCs) sensitized with Z907 dye. Structural, morphological, optical, and textural characterizations (XRD, Raman, SEM, DRS, BET, UV–vis, and FTIR) revealed systematic Ga-induced modifications of the anatase TiO_2_ structure and progressive reduction in crystallite and particle sizes, together with a significant increase in specific surface area. Moderate nominal Ga content enhanced dye adsorption and visible-light harvesting without substantially modifying the TiO_2_ bandgap. Photovoltaic performance, evaluated by current density–voltage (J-V) and open-circuit voltage decay (OCVD) measurements, showed that the photoanode fabricated with 4% nominal Ga content performed best, achieving an approximately 60% enhancement in power conversion efficiency compared with undoped TiO_2_ devices. The improved photovoltaic response is attributed to enhanced light harvesting, improved charge injection, and reduced recombination losses, driven by favorable modification of the trap-state distribution and interfacial charge-transfer processes. In contrast, higher nominal Ga contents increased the distribution of deep trap states and inefficient semiconductor-dye coupling, reducing device performance. Stability tests conducted over 30 days confirmed that TiO_2_-based photoanodes with a nominal 4% Ga content exhibited the best long-term photovoltaic stability. The findings demonstrate that controlled Ga doping is an effective strategy for tailoring the physicochemical and electronic properties of TiO_2_ photoanodes and improving DSSC performance.

## Graphical abstract

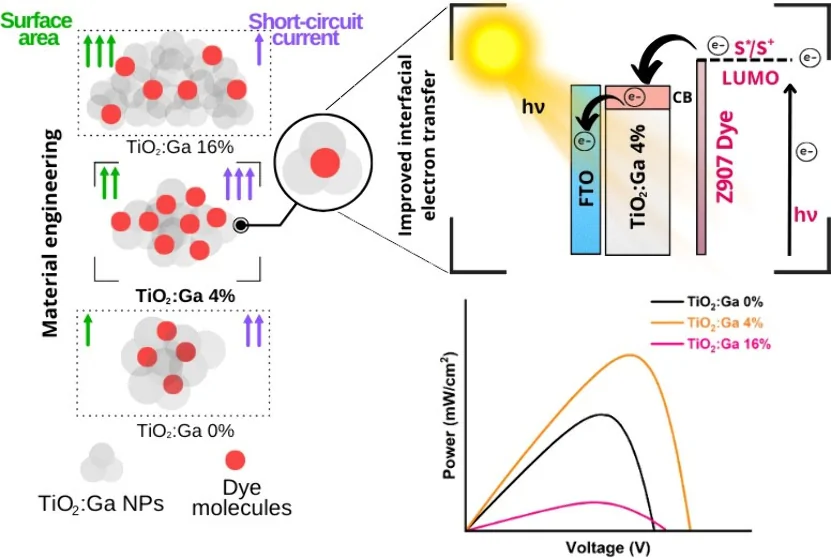

## 1 Introduction

The growing environmental impact of fossil fuel consumption has accelerated the development of renewable energy technologies; one of them is photovoltaic (PV) systems, which directly convert solar radiation into electricity.^1,2^ PV technologies include crystalline silicon devices, thin-film systems such as CdTe, CIGS, and GaAs, and emerging third-generation approaches including dye-sensitized solar cells (DSSCs), organic solar cells, quantum-dot devices, and perovskite solar cells.^3–7^ Among these, DSSCs are notable for their simple architecture, low fabrication cost, and functional versatility. They typically comprise a dye-sensitized mesoporous TiO_2_ photoanode, a redox electrolyte, and a platinized counter electrode.^4,8–10^

DSSCs rely on strong visible-light harvesting, efficient charge separation, relatively fast charge transport, and slow recombination.^8–10^ In DSSCs, light absorption and electron transport occur in different materials, unlike in single-crystal solar cells.^10^ This allows the use of different semiconductor nanostructures as photoanodes to increase the effective surface area for dye adsorption, reduce charge recombination, and improve charge transport. Consequently, optimizing photoanode performance requires fine-tuning both electron kinetics (transport and recombination) and surface chemical reactivity. To achieve this, elemental and composite doping strategies have emerged as one of the most effective approaches for engineering the electronic structure and surface features of TiO_2_ photoanodes.^11,12^ Various dopants, ranging from transition metals and nonmetals (such as Cu, S, and Cu/S codoping) to carbon nanocomposites and plasmonic metal modifications, have been extensively explored to reduce charge recombination and improve short-circuit current density (*J*_SC_) and open-circuit voltage (*V*_OC_).^11–13^ Furthermore, aliovalent cationic doping (Ga^3+^ for Ti^4+^) induces lattice strain and oxygen vacancies (*V*_O_), which substantially boosts the density of surface hydroxyl (−OH) groups and active surface sites.^14^ In environmental chemistry and photocatalysis, these surface −OH sites act as effective hole traps, generating highly reactive hydroxyl radicals (•OH) for pollutant degradation.^14^ In photoanodes, enhanced surface hydroxylation alters TiO_2_ surface acidity, promoting stronger anchoring of sensitizing dyes (via carboxylate linkages) and suppressing interfacial recombination.

Chandiran et al. reported that Ga-doped TiO_2_ photoanodes achieve notable improvements in open-circuit voltage and short-circuit current density compared to their undoped counterparts.^15^ These improvements are partly attributed to the favorable incorporation of Ga^3+^ into Ti^4+^ lattice sites, facilitated by their ionic radii (Ga^3+^ = 0.62 Å and Ti^4+^ = 0.68 Å).^14,16^ More importantly, Ga incorporation can modify both the electronic structure and defect-state distribution of TiO_2_, influencing the charge-generation, transport, and recombination processes that affect DSSC performance. Since the lower edge of the TiO_2_ conduction band is dominated by Ti 3d states, substitution of Ti^4+^ by a foreign cation can perturb the conduction band structure and consequently affect the open-circuit voltage (*V*_OC_) and the injection of photogenerated electrons.^17^ Changes in the density and distribution of defect states can additionally influence electron trapping and detrapping, carrier lifetime, and recombination kinetics, thereby affecting both *V*_OC_ and *J*_SC_.^18^ As electron transport in mesoporous TiO_2_ involves trap-mediated hopping and detrapping, modification of these states may also alter electron mobility and collection efficiency.^19^

Despite the growing interest in microwave-assisted synthesis as a rapid alternative to conventional thermal processing, relatively few studies have systematically correlated microwave-induced changes in the structural, surface, and electronic properties of doped TiO_2_ with resulting DSSC performance.^20^ In this work, we employed a microwave-assisted route to synthesize mesoporous anatase Ga-doped TiO_2_ nanoparticles for Z907-sensitized photoanodes. We examined how Ga incorporation affects crystal structure, morphology, surface area, dye loading, and interfacial charge recombination. Using open-circuit voltage decay (OCVD) analysis and complementary photovoltaic measurements, we show that an optimum nominal Ga content balances surface-related and electronic effects, yielding an approximately 60% increase in power conversion efficiency (PCE) and improved operational stability over 30 days.

## 2 Experimental Section

### 2.1 Synthesis of Undoped and Ga-Doped TiO_2_ Nanoparticles

Undoped and Ga-doped TiO_2_ nanoparticles with nominal Ga contents of 0, 1, 2, 4, 8, and 16 mol % were synthesized by the microwave-assisted sol–gel method. Titanium tetraisopropoxide (TTIP, 97%, Sigma-Aldrich) and gallium­(III) nitrate hydrate (Ga­(NO_3_)_3_•XH_2_O 99.9%, Alfa Aesar) were used as Ti and Ga precursors, respectively, while absolute ethanol (C_2_H_5_OH, 99.9%, J.T. Baker) served as the solvent. In a typical synthesis, 3.6 mL of TTIP was dissolved in 240 mL of ethanol, and 1 mL of nitric acid (HNO_3_, 65.8%, J.T. Baker) was added to suppress premature hydrolysis. The desired amount of Ga precursor (0–16 mol %) was added, and the mixture was stirred for 24 h to ensure complete homogenization. Subsequently, 15 mL of this precursor solution was mixed with 15 mL of ethanol and stirred for 15 min. Controlled hydrolysis and condensation were induced by adding ammonium hydroxide (NH_4_OH, 28.7%, Fermont) to adjust the pH to ∼7.0,^21^ followed by an additional 30 min of stirring. Microwave irradiation was carried out in a 900 W (Panasonic NN-SB428SRUH) domestic microwave oven operated at 180 W for 3 min using pulse cycles (8 s on/25 s off, four cycles with 10 min intervals). The maximum reactor temperature was routinely measured using a calibrated infrared thermometer (Milwaukee 2267-20, accuracy ±2 °C), reaching approximately 135 °C.^22^ To ensure strict experimental reproducibility in the absence of continuous in situ thermal feedback, all physical and operational parameters, including solvent volume (30 mL), reaction vessel geometry and position, nominal applied power (180 W), and pulse sequencing, were rigorously standardized across all synthesis runs. Figure S1 (Supporting Information) shows the reactor temperature profile during microwave-assisted synthesis. After cooling to room temperature, the precipitates were collected by centrifugation, washed thoroughly with ethanol and deionized water, and dried at 70 °C for 24 h to obtain fine powders. Each synthesis was repeated at least six times to ensure reproducibility. The samples prepared with nominal 0, 1, 2, 4, 8, and 16 mol % of Ga were designated as TiO_2_:Ga-0, TiO_2_:Ga-1, TiO_2_:Ga-2, TiO_2_:Ga-4, TiO_2_:Ga-8, and TiO_2_:Ga-16, respectively.

### 2.2 Fabrication of DSSC Components

A compact TiO_2_ layer was deposited onto fluorine-doped tin oxide (FTO) glass substrates (20 mm × 15 mm) via spray pyrolysis at 350 °C, using titanium diisopropoxide bis­(acetylacetonate) (75%) as the precursor. The photoanode was prepared by dispersing 200 mg of TiO_2_ nanopowder in 0.450 mL of deionized water, 30 μL of acetylacetone, and one drop of Triton X-100.^23,24^ The mixture was stirred for 10 min, ultrasonicated, and subsequently stirred for 24 h to ensure a homogeneous and stable dispersion. The resulting paste was deposited onto the FTO substrates using a manual doctor-blade technique. The active area of the fabricated solid-state thin films (0.35 cm^2^) was defined using a shadow mask during photovoltaic measurements. The dye solution was prepared by dissolving 0.375 mM Z907 dye (C_42_H_52_N_6_O_4_RuS_2_, Greatcell Solar) in a mixed solvent of acetonitrile and *tert*-butyl alcohol (1:1, v/v%; total volume 30 mL), followed by stirring for 24 h. To avoid any surface modification of the TiO_2_–Ga electrodes, a simple electrolyte was used in this study, consisting of 0.6 M 1-butyl-3-methylimidazolium iodide and 0.03 M iodine dissolved in an acetonitrile/valeronitrile mixture (85:15, v/v%).^25^ Counter electrodes were fabricated by depositing two drops of a 5 mM chloroplatinic acid solution (H_2_PtCl_6_, 8 wt %, Sigma-Aldrich) in 2-propanol ((CH_3_)_2_CHOH, 95%, Sigma-Aldrich) onto FTO substrates, followed by annealing at 450 °C for 1 h.^25^ The DSSCs were assembled by sandwiching the dye-sensitized photoanode and the Pt-coated counter electrode using a 25 μm-thick Surlyn spacer (Dupont grade 1702). The electrolyte was introduced through predrilled holes (∼2 mm in diameter), which were subsequently sealed to ensure proper encapsulation.

### 2.3 Materials Characterization

X-ray diffraction (XRD) was employed to assess the phase purity, crystal structure, and structural parameters of the films prepared from the synthesized undoped and Ga-doped TiO_2_ nanoparticles. The diffraction patterns were recorded using a PANalytical Empyrean diffractometer with Co Kα radiation (λ = 1.7889 Å) at room temperature with a step size of 0.02° over a 2θ range from 10 to 90 deg. The structural parameters were determined by Rietveld refinement of the XRD patterns using BGMN through the Profex graphical user interface (version 5.7.1), employing a structural model with the tetragonal *I4*_1_/*amd* space group for anatase TiO_2_ to determine the lattice parameters and crystallite size.^26^ To support the Rietveld refinement, we used the crystallographic information file (CIF) for TiO_2_ anatase (ICSD no. 9852) as the reference. The crystallite size (*D*) was obtained from the crystallite-size broadening parameter refined in BGMN/Profex, while the microstrain (*ε*) was determined from the strain-broadening parameter *k*2, according to $\varepsilon =\sqrt{k2}$ .^26^

The Raman measurements were performed using a Horiba LabRAM HR spectrometer equipped with a thermoelectrically cooled charge-coupled device (CCD) detector with spatial and spectral resolutions of 20, 5 and 2 μm, and ± 0.2 to ± 0.5 cm^–1^, respectively. The spectra were collected at room temperature using a He–Ne laser (λ = 632.8 nm) as the excitation source. The morphology and microstructure of the samples were examined using a field-emission scanning electron microscope (FE-SEM, JEOL JSM-7800F) operated at an accelerating voltage of 5.0 kV. Elemental composition was analyzed by energy-dispersive X-ray spectroscopy (EDS) using an Oxford Instruments X-Max detector attached to the FE-SEM system operated at 15 kV.

Optical properties of the samples were investigated by diffuse reflectance spectroscopy (DRS) in the UV–vis region using a Varian Cary 5000 UV–vis–NIR spectrophotometer equipped with an integrating sphere accessory. The reflectance data were transformed using the Kubelka–Munk function to evaluate absorption characteristics and estimate the optical bandgap energy. Specific surface area and pore characteristics were determined by nitrogen adsorption–desorption measurements using the Brunauer–Emmett–Teller (BET) method with a BELSORP Mini-II (BEL, Japan) sorptometer. Prior to analysis, the samples were degassed under vacuum (∼10 Pa) at 90 °C for 15 h to remove adsorbed species. The BET surface area was calculated from the adsorption isotherms, while the pore size distribution was derived using the Barrett–Joyner–Halenda (BJH) model. To identify the characteristic functional groups and active vibrational modes of the sensitized films, as well as to evaluate the interactions between the dye molecules and the surface of the undoped and Ga-doped TiO_2_ photoanodes, Fourier-Transform Infrared (FTIR) spectra were recorded on a Cary 630 FTIR spectrometer over the range of 600–4000 cm ^–1^.

UV–vis absorption spectroscopy was used to quantify the amount of dye desorbed from the sensitized photoanodes (0.35 cm^2^). The dye molecules were desorbed from the sensitized films in 5 mL of 0.1 NaOH water/ethanol solution (50:50, v/v%) for 2 h. The dye concentration in the NaOH desorption solution was quantified using a UV–vis absorption spectrophotometer (Shimadzu UV-3101PC) over a wavelength range of 300–900 nm.

### 2.4 Device Characterization

The photovoltaic performance of the fabricated DSSCs based on undoped and Ga-doped TiO_2_ photoanodes was systematically evaluated under standard Air Mass (AM1.5) illumination at an incident power density of 100 mW cm^–2^. The current density–voltage (*J*–*V*) characteristics were measured using a home-built photovoltaic characterization setup equipped with a xenon lamp and a Keithley 2450 source meter unit (SMU). The irradiance at the device plane was adjusted to one-sun-equivalent intensity (100 mW cm^–2^) using a calibrated Si reference cell. Photovoltaic parameters were averaged across at least 5 different devices.

The open-circuit voltage decay (OCVD) measurements were carried out under pulsed white-light excitation, using a mounted white LED source (MWHL3, Thorlabs) controlled by a function generator (AFG1022, Tektronix) to regulate the illumination amplitude and pulse duration. During the measurements, the devices were maintained under open-circuit conditions and connected to a digital storage oscilloscope (TBS1102B, Tektronix), which recorded the temporal evolution of the open-circuit voltage, *V*_OC_(*t*), immediately after the illumination was switched off. The resulting voltage-decay profiles provide direct insight into electron lifetimes and recombination processes at the photoanode–electrolyte interface.

## 3 Results and Discussion

The crystalline, morphological, optical, and textural properties of the synthesized materials were systematically analyzed using XRD, Raman spectroscopy, SEM, DRS/UV–vis spectroscopy, and BET analyses. The photovoltaic performance of the fabricated DSSCs was evaluated through current density–voltage (*J*–*V*) measurements and OCVD analysis, including stability assessment over 30 days.

Figure 1 presents the refined XRD patterns of undoped and Ga-doped TiO_2_ films. Rietveld refinements yielded weighted profile *R*-factor (*R*_wp_) values ranging from 3.97% to 5.67%, while the goodness-of-fit (GoF) values ranged from 0.98 to 1.35, showing good agreement between the experimental data and fitted results. All reflections were indexed to TiO_2_ with a tetragonal crystal structure, indicating that Ga incorporation does not alter the host lattice. No additional reflections associated with secondary phases or impurities were detected. A gradual decrease in diffraction peak intensity, accompanied by peak broadening, is observed as Ga concentration increases, suggesting reduced crystallinity and increased lattice disorder.^27^ Although Ga^3+^ (0.62 Å) and Ti^4+^ (0.68 Å) have comparable ionic radii, partial substitution of Ti^4+^ by Ga^3+^ requires local charge compensation, for which oxygen vacancies and/or interstitial defects have been proposed as plausible mechanisms.^28–30^ These defects may contribute to microstrain and disrupt the periodicity of the TiO_2_ lattice, leading to peak broadening and reduction in crystallite size. To quantitatively evaluate these structural changes, the lattice parameters (*a* and *c*), unit-cell volume, crystallite size, and microstrain were obtained from Rietveld refinement. The refined structural parameters and their corresponding standard errors are summarized in Table 1.

**Figure 1:**
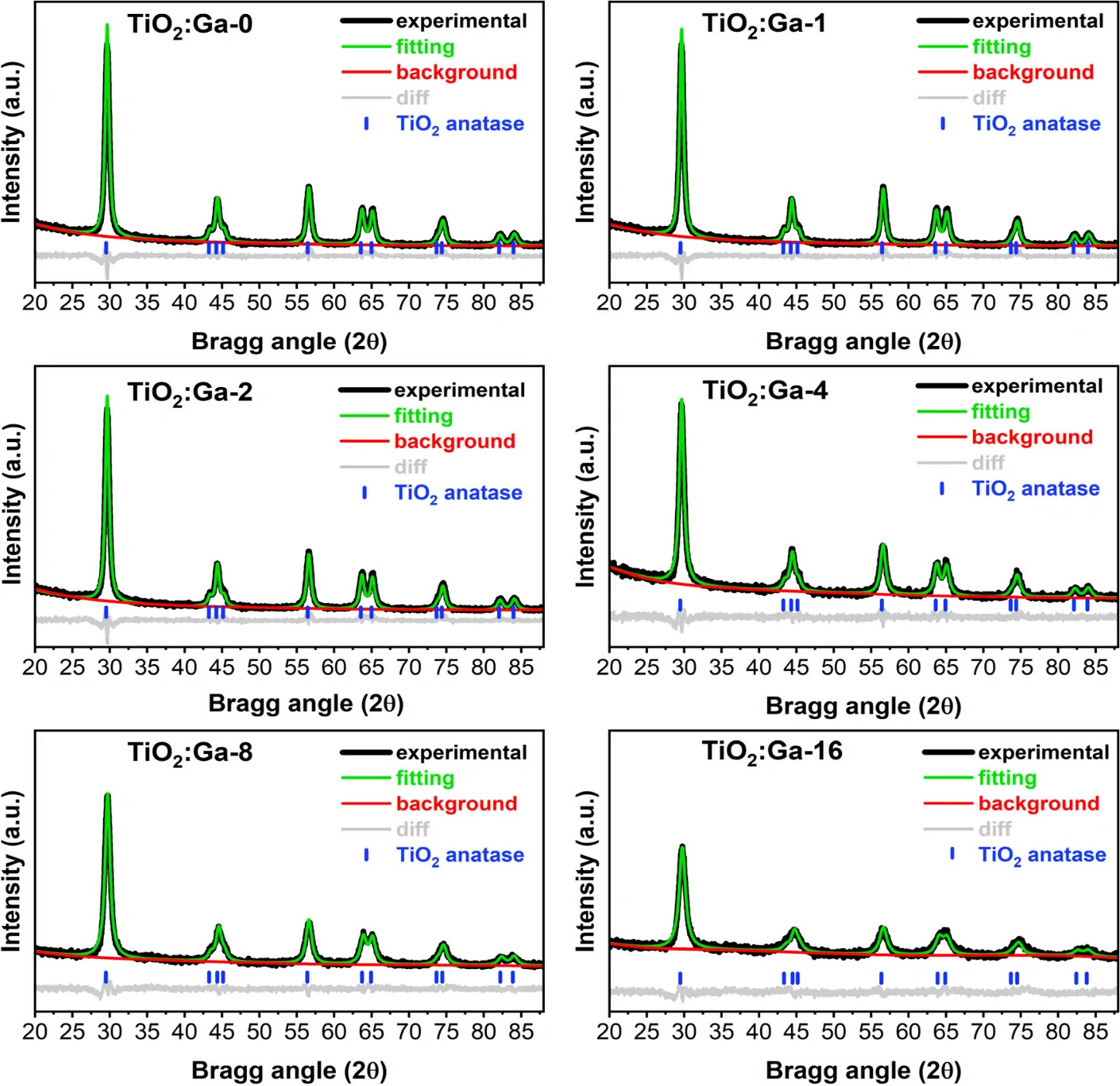
Rietveld analysis output profiles of XRD patterns of undoped and Ga-doped TiO_2_ films with different nominal Ga contents.

**Table 1:** Rietveld Refinement Quality Indicators and Structural Parameters (Lattice Parameters, Unit-Cell Volume, Crystallite Size, and Microstrain) For Undoped and Ga-Doped TiO_2_ Films^a^

metric/parameter	TiO_2_:Ga-0	TiO_2_:Ga-1	TiO_2_:Ga-2	TiO_2_:Ga-4	TiO_2_:Ga-8	TiO_2_:Ga-16
*R*_wp_	5.47	5.33	5.67	5.05	4.22	3.97
*R*_exp_	4.31	4.47	5.06	5.14	4.02	3.09
χ^2^	1.19	1.14	1.22	0.96	1.10	1.13
GoF	1.35	1.11	1.14	0.98	1.04	1.06
*a* (Å)	3.78528(2)	3.78501(2)	3.78234(1)	3.78228(3)	3.78130(2)	3.780(1)
*c* (Å)	9.51064(6)	9.51011(5)	9.50981(6)	9.50009(8)	9.48655(8)	9.450(2)
V (Å^3^)	136.345(1)	136.245(1)	136.048(2)	135.904(2)	135.640(2)	135.081(4)
*D* (nm)	15.875(4)	15.452(2)	15.231(2)	12.762(3)	11.171(2)	9.104(4)
*ε* (10^–3^)	1.386(13)	1.342(32)	1.312(31)	1.843(21)	2.199(12)	2.329(4)
ρ (g•cm^–3^)	3.800	3.822	3.822	3.894	3.897	3.915
Ti:4a _(*x*,*y*,*z*)_	0.0000	0.0000	0.0000	0.0000	0.0000	0.0000
O:8e _(*z*)_	0.2180	0.2180	0.2180	0.2180	0.2180	0.2180

a V = unit cell volume, *D* = average crystallite size, ε = microstrain, ρ = crystallographic density, Ti:4a and O:8e = Wyckoff positions.

The instrumental contribution to peak broadening was evaluated using a standard LaB_6_ reference material measured under the same experimental conditions as the samples. An instrumental fwhm (β_inst_) of approximately 0.023° (2θ) was obtained. The measured peak broadening (β_exp_) was corrected for the instrumental contribution by quadratic subtraction according to

1 $$\beta_{\mathrm{sample}}=\sqrt{\beta_{\exp}^{2}-\beta_{\mathrm{inst}}^{2}}$$

where β_sample_ represents the sample-related peak broadening. The instrumental contribution was accounted for in the profile analysis during Rietveld refinement using BGMN through the Profex interface, allowing the sample-related broadening associated with crystallite size and microstrain to be evaluated.

As summarized in Table 1, the lattice parameters and unit cell volume vary slightly as Ga concentration increases, with these changes becoming more pronounced at higher Ga contents (≥4 mol % nominal). These variations are attributed to lattice distortion and defect formation associated with Ga-induced structural modification. In contrast, the average crystallite size decreases markedly with increasing dopant content, particularly for samples with nominal 4, 8, and 16% Ga. This reduction is associated with the substitutional incorporation of Ga^3+^ into the TiO_2_ lattice, which inhibits crystal growth and enhances lattice strain, thereby limiting grain coarsening.^29^

The crystallographic parameters extracted from Rietveld refinement indicate noticeable changes in the lattice parameter *c* with increasing nominal Ga content, as also reflected in the crystallite size. Figure S2 (Supporting Information) shows the variation in the lattice parameter *c* and the average crystallite size, along with their respective error bars. Additional parameters, such as microstrain, show a slight decrease up to 2%, followed by a marked increase with increasing nominal Ga content, consistent with Ga-induced structural modification and increased strain in the TiO_2_ anatase lattice. Furthermore, the Wyckoff (Ti:4a, O:8e) positions remain virtually unchanged across all samples, indicating that Ga incorporation does not alter the atomic positions of the anatase phase. As the ionic radius of Ga^3+^ is about 9% smaller than that of Ti^4+^, the gradual decrease in unit cell volume with increasing Ga incorporation provides structural evidence for the substitutional incorporation of Ga^3+^ at Ti^4+^ sites in the TiO_2_ lattice.

Figure 2 presents the Raman spectra of the undoped and Ga-doped TiO_2_ films. All observed vibrational modes correspond to phase-pure anatase TiO_2_, with no detectable impurity-related bands, in full agreement with XRD results. The characteristic Raman-active modes of anatase were identified at around 144 (E_g_), 197 (E_g_), 398 (B_1g_), 514 (B_1g_), and 639 (E_g_) cm^–1^.^27^ Figure 2b shows a magnified view of the dominant E_g_ mode at ∼144 cm^–1^, and Table 2 summarizes the corresponding peak positions and fwhm values. A slight blue shift of this mode toward higher wavenumbers is observed with increasing Ga content, accompanied by progressive peak broadening and reduced intensity. These changes indicate increased lattice disorder and structural distortion induced by Ga incorporation.^15,28,30^ The gradual decrease in the intensity of the characteristic anatase modes (particularly the main E_g_ mode) is consistent with Ga-induced structural modification and increased disorder in the TiO_2_ lattice. On partial substitution of Ti^4+^ by Ga^3+^, local charge compensation may generate defect states, including oxygen vacancies, which can dampen lattice phonon vibrations and disrupt long-range translational symmetry. The progressive peak broadening (increased fwhm) is attributed to phonon confinement from Ga-induced crystallite size reduction (consistent with our XRD findings in Table 1) and a shortened phonon lifetime due to scattering at local lattice distortions and defect sites.^31^ Consequently, the reduction in phonon coherence length leads to peak broadening and intensity attenuation. These features are consistent with the combined effects of lattice strain and reduced crystallite size, while oxygen-vacancy-related disorder may also contribute, in agreement with the trends observed in XRD analysis.^27,30,31^

**Figure 2:**
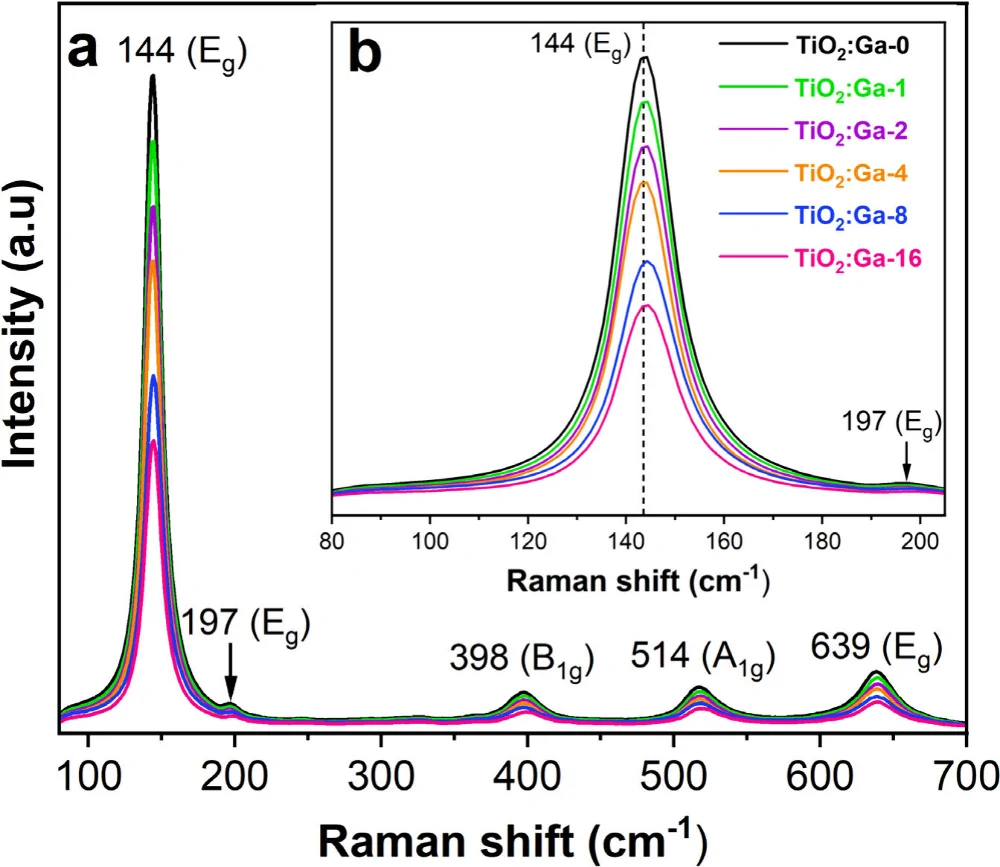
(a) Raman spectra of the undoped and Ga-doped TiO_2_ films, and (b) enlarged view of the E_g_ mode (∼144 cm^–1^) showing peak shift and broadening with increasing nominal Ga content.

**Table 2:** Evolution of the Raman E_g_ Mode Position and FWHM as a Function of Nominal Ga Content in TiO_2_ Films

Film sample (nominal Ga content)	Peak position (cm^–1^)	fwhm (cm^–1^)
0%	144.01 ± 0.04	13.86 ± 0.53
1%	144.10 ± 0.02	14.62 ± 0.55
2%	144.14 ± 0.06	14.87 ± 0.54
4%	144.17 ± 0.04	15.76 ± 0.22
8%	144.22 ± 0.03	18.06 ± 0.66
16%	144.29 ± 0.02	22.34 ± 1.03

Figure 3 shows representative SEM images of the undoped and Ga-doped TiO_2_ films. All samples exhibited quasi-spherical nanoparticle morphology, forming continuous, homogeneous films with slight agglomeration and relatively narrow size distributions. No visible cracks or discontinuities are observed, indicating good film integrity across all compositions. Particle size decreases gradually as the Ga concentration increases. Quantitative analysis (Table 3) reveals that the average particle size decreases from approximately 144 nm for the undoped film to ∼70 nm for the TiO_2_ film fabricated with 16% nominal Ga content. In addition to the reduction in average size, the particle-size distribution becomes narrower at higher doping levels, indicating improved uniformity of the nanocrystalline network. This trend is consistent with the crystallite-size reduction observed in XRD results, confirming that Ga incorporation effectively suppresses TiO_2_ grain growth by introducing lattice strain and defect sites.^28,29^

**Figure 3:**
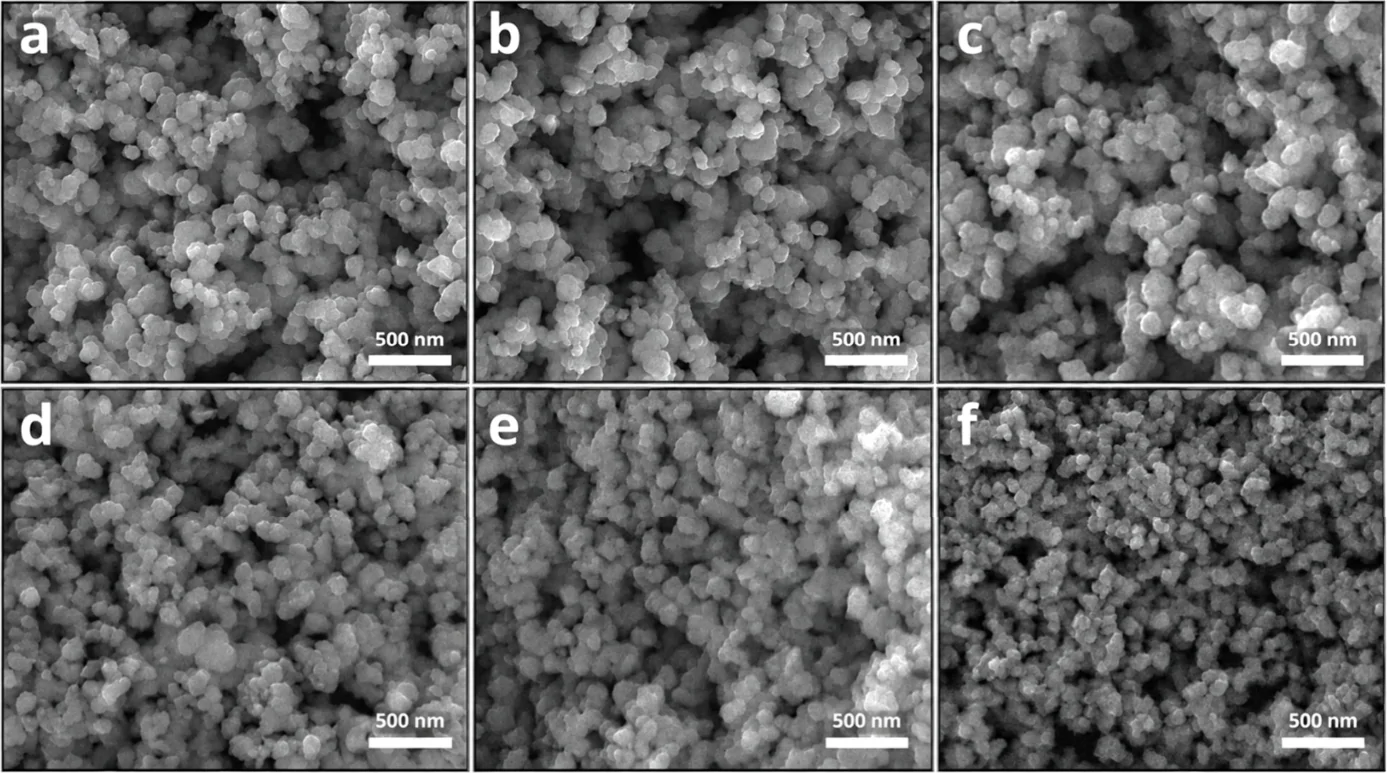
Representative SEM images of TiO_2_ films prepared with nominal Ga contents of (a) 0%, (b) 1%, (c) 2%, (d) 4%, (e) 8%, and (f) 16%. All samples exhibit a quasi-spherical morphology, with particle size decreasing as Ga content increases.

**Table 3:** Mean Particle Size and Film Thickness of the Undoped and Ga-Doped TiO_2_ Films

Film sample (Nominal Ga content)	Mean size (nm)	Mean thickness (μm)
0%	144.2 ± 50.1	11.8 ± 0.5
1%	142.4 ± 47.6	11.5 ± 0.6
2%	140.9 ± 44.5	12.1 ± 0.5
4%	119.5 ± 42.3	11.9 ± 0.5
8%	105.8 ± 28.9	14.3 ± 0.7
16%	70.4 ± 21.8	11.8 ± 1.3

Film thickness was determined by stylus profilometry using the step-height method, in which a well-defined step was generated by locally removing the TiO_2_ layer to expose the underlying FTO substrate.^8^ The films were deposited using a manual doctor-blade technique, widely employed in DSSC fabrication.^8,32^ Thickness measurements were performed at multiple positions across representative areas (∼0.7 cm^2^) to evaluate both thickness and lateral uniformity. The measured thickness values ranged from 11 to 14 μm, consistent with typical mesoporous photoanodes reported in the literature for DSSC applications.^32–35^ The observed variations are primarily attributed to differences in the rheological properties of the undoped and Ga-doped TiO_2_ pastes. Specifically, dopant incorporation and solvent composition influence the viscosity and interparticle interactions, which in turn affect film formation. In manual doctor-blade deposition, such rheological variations are further amplified by minor fluctuations in coating parameters, including blade pressure, coating speed, and solvent evaporation rate, leading to measurable differences in the final film thickness.^35^

Table 4 presents the elemental compositions (at.%) of undoped and Ga-doped TiO_2_ films obtained by EDS analysis. To compare the nominal composition with the EDS-estimated results on the same basis, we expressed the EDS-estimated Ga contents as [Ga (at.%)/Ti (at.%)] × 100%. The resulting Ga contents were estimated to be 1.51, 2.16, 3.70, 9.22, and 19.63% for the samples prepared with nominal 1, 2, 4, 8, and 16 mol %, respectively. In general, the EDS-estimated fractional Ga contents follow the nominal compositional trend, with the closest agreement observed for the intermediate compositions. The remaining deviations may reflect differences in the hydrolysis and condensation kinetics of the Ti and Ga precursors during the microwave-assisted sol–gel process, as well as the local and semiquantitative nature of EDS analysis. Accordingly, EDS provides an experimental estimate of the Ga content present in the processed TiO_2_-based films, rather than the nominal precursor composition.^27,31^ Finally, the increase in carbon content detected by EDS (Table 4) is attributed to residual carbonaceous species originating from the paste preparation and processing steps.

**Table 4:** Elemental Composition (at.%) of Undoped and Ga-Doped TiO_2_ Films Determined by EDS, Together with the Nominal Ga/Ti Mole Fraction in Percentage and EDS-Estimated Ga/Ti Mole Fraction in Percentage^a^

Parameter	Undoped TiO_2_	Ga-doped TiO_2_				
Nominal Ga/Ti molar ratio (mol %)	0	1	2	4	8	16
EDS Elemental Composition (at.%)						
Ti	21.3	19.9	18.5	16.2	14.1	10.7
O	71.4	71.7	71.8	72.8	73.2	73.9
Ga		0.3	0.4	0.6	1.3	2.1
C	7.2	8.1	9.3	10.4	11.4	13.3
**EDS-Estimated Ga/Ti Ratio (%)**	**0**	**1.51**	**2.16**	**3.70**	**9.22**	**19.63**

a Both the nominal and EDS-estimated Ga/Ti fractions were estimated as [Ga (at.%)/Ti (at.%)] × 100%.

The optical properties of the undoped and Ga-doped TiO_2_ films were investigated by DRS in the wavelength range of 200–1000 nm after annealing at 500 °C for 2 h. The undoped TiO_2_ films exhibited a sharp absorption edge at approximately 390 nm, characteristic of anatase TiO_2_.^28,30^ With increasing Ga content, a gradual shift of the absorption edge toward shorter wavelengths (blue shift) was observed, indicating a modification of the electronic structure.

To quantitatively analyze the optical behavior, the reflectance data were transformed using the Kubelka–Munk (K–M) function, which relates diffuse reflectance to the absorption coefficient through the relation:

2 $$F\left(R_{\alpha }\right)=\frac{{\left(1-R_{\alpha }\right)}^{2}}{2R_{\alpha }}$$

where *R*_α_ denotes the diffuse reflectance of an optically thick sample relative to a reference standard. The optical bandgap energies were estimated from the K–M plots as a function of photon energy (Figure 4), assuming an indirect transition for TiO_2_.^30,36^ A systematic but small increase in bandgap energy is observed as Ga concentration increases, consistent with a progressive blue shift of the absorption edge. This behavior is primarily attributed to the inhibition of crystallite growth and stabilization of the anatase phase, both of which influence the electronic structure near the band edges. Furthermore, possible aliovalent substitution of Ti^4+^ by Ga^3+^ and the associated formation of oxygen-related defects can modulate the band edge without introducing dominant midgap states. As a result, the optical absorption remains governed by intrinsic band-to-band transitions, leading to a systematic widening of the bandgap.^37^ This trend is consistent with previous reports on Ga-doped TiO_2_ systems, where similar blue shifts have been attributed to size reduction, lattice distortion, and defect-mediated electronic effects.^27,30,36^

**Figure 4:**
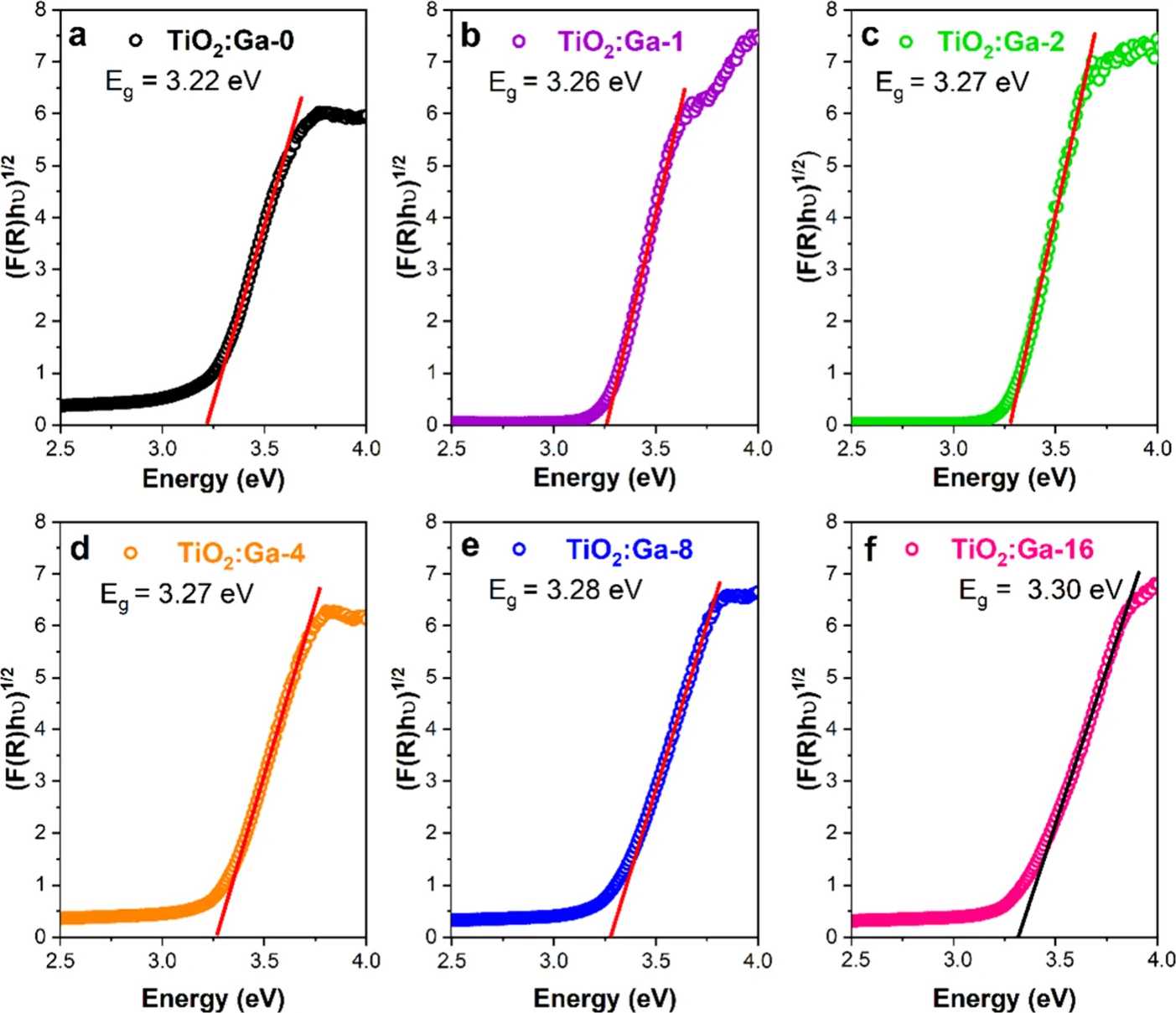
Kubelka–Munk-transformed diffuse reflectance spectra of the films prepared with nominal Ga contents of (a) 0%, (b) 1%, (c) 2%, (d) 4%, (e) 8%, and (f) 16%, used to evaluate indirect optical bandgap energies.

The textural properties of undoped and Ga-doped TiO_2_ samples were evaluated by N_2_ adsorption–desorption measurements at 77 K. The specific surface area was determined using the Brunauer–Emmett–Teller (BET) method from the linear region of the adsorption isotherm within the relative pressure (p/p_0_) range of 0.05–0.30. The pore-size distribution and pore-related parameters were determined using the Barrett–Joyner–Halenda (BJH) method. The corresponding textural parameters are summarized in Table 5, including the BET surface area, total pore volume, and average pore diameter. All the samples exhibit type IV isotherms with H3-type hysteresis loops, characteristic of mesoporous materials with slit-shaped pores.^28,38,39^ The pronounced hysteresis and increased nitrogen uptake at higher relative pressures (*p*/*p*_0_ > 0.8) further confirm the presence of a well-developed mesoporous structure. Notably, Ga incorporation significantly enhances the adsorption capacity, particularly in samples fabricated with 8% and 16% nominal Ga contents, indicating increased pore volume and improved accessibility at higher pressures.

**Table 5:** BET Surface Area, Pore Volume, and Average Pore Diameter of Undoped and Ga-Doped TiO_2_ Samples Obtained from N_2_ Adsorption–desorption Isotherms

Sample (Nominal Ga content)	*a*_s_BET (m^2^ g^–1^)	*V*_m_ (cm^3^ g^–1^)	Mean pore diameter (nm)
0%	307.2	0.257	3.03
1%	310.1	0.258	3.05
2%	312.3	0.258	3.10
4%	336.7	0.260	3.33
8%	434.1	0.504	4.48
16%	544.4	0.543	4.61

A clear trend of increasing BET surface area with increasing Ga content is observed, rising from ∼307 m^2^ g^–1^ for undoped TiO_2_ to 544 m^2^ g^–1^ for the sample fabricated with 16% nominal Ga content. This increase is accompanied by a substantial rise in pore volume and a moderate increase in the average pore diameter, reaching 4.5 nm for the highly doped samples. The corresponding BJH pore-size distributions (Figure S3, Supporting Information) exhibit a narrow distribution with a dominant peak in the 2.18–2.45 nm range. The maximum shifts slightly toward smaller pore diameters with increasing Ga content (from 2.45 nm for undoped TiO_2_ to 2.18 nm for the sample fabricated with 16% nominal Ga content), while the overall mesoporous character is preserved. Concurrently, the total pore volume and BJH surface area increase progressively, consistent with the higher BET specific surface area measured for the Ga-doped samples. These values are consistent with those reported for mesoporous TiO_2_ systems with nanostructured architectures.^40–42^ The increase in surface area and porosity is consistent with the reduction in crystallite size observed by XRD and the smaller particle size observed by SEM. The incorporation of Ga hinders crystal growth during synthesis, favoring the formation of finer nanoparticles that assemble into less densely packed aggregates with a larger volume of interconnected interparticle mesopores. These structural features plausibly explain the progressive increase in BET surface area with increasing Ga content. This enhanced surface area and porosity benefit DSSC applications by facilitating higher dye loading, improved electrolyte diffusion, and efficient charge transport within the photoanode.^8,30^

The experimentally determined BET surface areas exceed those predicted by the simplified geometric relationship SSA = 6/(ρ •*d*), which assumes smooth, isolated, nonporous spherical particles. These assumptions are not applicable to the present Ga-doped TiO_2_ powders. XRD analysis reveals nanocrystalline domains, Raman spectroscopy indicates increasing structural disorder with Ga incorporation, and BJH analysis shows a progressively more developed mesoporous network as pore volume increases. Consequently, nitrogen adsorption probes not only the external particle surfaces but also the accessible internal surfaces associated with interconnected interparticle mesopores, resulting in substantially higher BET surface areas than those predicted for ideal compact spheres.

The UV–Vis absorption spectra of Z907-sensitized TiO_2_ films with different nominal Ga contents (Figure 5a) exhibit a broad absorption extending from the UV to the visible region, with an absorption onset around 380–390 nm, consistent with the intrinsic band edge of anatase TiO_2_.^30,36^ In the visible region, the spectra are dominated by the metal-to-ligand charge-transfer (MLCT) transitions of the Z907 dye, centered at ∼610 nm. However, the absorption profile gradually broadens as Ga content increases. This effect does not correspond to a simple shift of the dye absorption band; rather, it results from the superposition of multiple spectral contributions. The primary contribution stems from the anchored Z907 dye molecules, while a secondary component arises from the TiO_2_-based matrix itself. This secondary component becomes more pronounced at higher doping levels and is associated with defect-induced electronic states, increased structural disorder, and enhanced light scattering caused by morphological changes.^42^ Consequently, the combined spectral response exhibits broader absorption without a distinct peak shift. Further evidence comes from the absorption spectra of unsensitized films, which show that the Ga-doped samples exhibit enhanced visible absorption compared with undoped TiO_2_ films. This additional absorption is attributed to sub-bandgap electronic states introduced by doping, typically associated with oxygen vacancies and localized Ti^3+^ species. These states overlap with the dye absorption region, producing an apparent red shift and an extended spectral response.^43^ At moderate doping levels, this enhancement can be attributed to both dye adsorption and changes in the interactions between dye molecules and the TiO_2_:Ga surface. The latter is supported by the FTIR analysis, which reveals changes in vibrational modes associated with the dye anchoring groups (COO^–^). In particular, the changes observed in the carboxylate-related vibrations suggest differences in the local environment and anchoring of the Z907 dye upon Ga incorporation.

**Figure 5:**
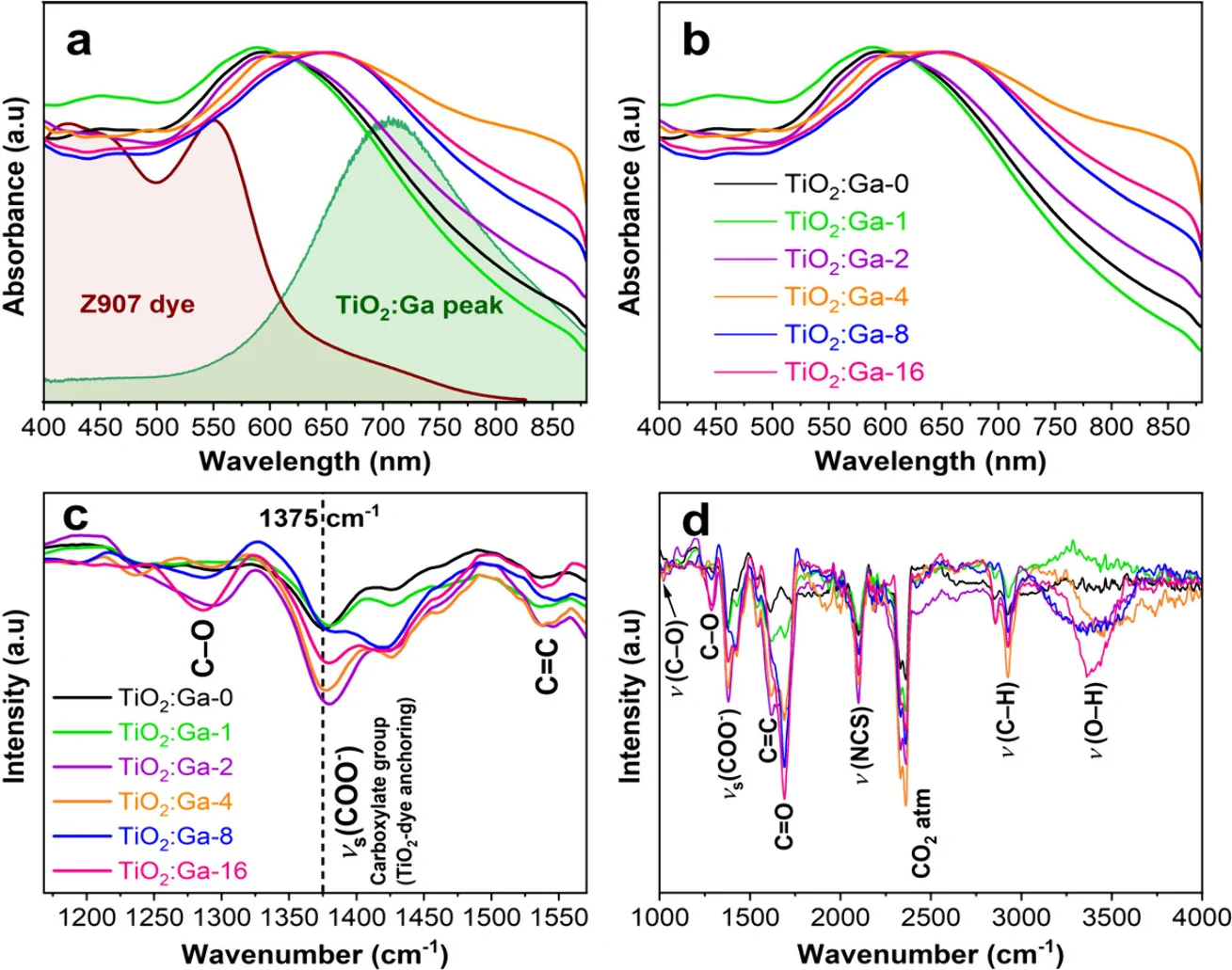
UV–vis absorption spectra of Z907 dye-sensitized photoanodes. (a) Deconvoluted absorption profile highlighting the individual contributions of the Z907 dye and Ga-doped TiO_2_ component. (b) Absorption spectra of TiO_2_ films with varying Ga content (0–16% nominal) after sensitization. (c) FTIR spectra in the 1200–1600 cm^–1^ region showing characteristic vibrations associated with dye anchoring. (d) Full-range FTIR spectra, indicating functional groups of Z907 dye and their interaction with the TiO_2_ surface.

FTIR spectra (Figure 5d) further confirm the chemical anchoring of the Z907 dye onto the TiO_2_ surface. Bands in the 1550–1600 cm^–1^ and 1380–1420 cm^–1^ regions correspond to asymmetric and symmetric carboxylate (COO^–^) stretching vibrations, indicating bidentate or bridging coordination to Ti surface sites.^43,44^ Additional bands associated with C=O stretching (1700–1730 cm^–1^) and C–O vibrations (1200–1300 cm^–1^) confirm the presence of organic functional groups originating from the Z907 dye molecules.^30,42^ Characteristic features related to the NCS ligand (∼2100 cm^–1^) and atmospheric CO_2_ (∼2350 cm^–1^) are also observed,^43–45^ while the broad band in the 3000–3600 cm^–1^ region corresponds to O–H stretching vibrations of the surface hydroxyl groups and adsorbed water molecules. The higher intensity of this band in the undoped sample suggests a greater degree of surface hydration. Importantly, the intensity of the COO^–^ bands increased for the sample prepared with nominal 2% and 4% Ga content, indicating enhanced dye adsorption and stronger interfacial binding. In contrast, at higher nominal Ga content (8% and 16%), the intensity of the NCS-related vibration decreases. Given the critical role of the NCS ligand in electron injection, this reduction suggests alterations in the dye coordination environment that may reduce charge-transfer efficiency. Overall, moderate Ga content (1–4% nominal) provides an optimal balance of increased surface area, enhanced dye adsorption, and favorable modification of the electronic structure. At higher doping concentrations, however, excessive defect formation and interfacial disorder may limit efficient dye–semiconductor coupling. These findings are consistent with BET and spectroscopic analyses and provide a strong basis for understanding the photovoltaic performance discussed in the following section.

To separate the contribution of the increased surface area from that of improved dye-TiO_2_ interactions, dye loading estimation was performed (Figure S4 and Figure S5). The dye concentration in the desorption solutions was quantified according to the Lambert–Beer Law, using the relation:

3 $$c=\frac{A}{\varepsilon l}$$

where *c* is the dye concentration, *A* is the absorbance, ε is the molar extinction coefficient, and *l* is the optical path length. The absorbance measured at 512 nm was used for the calculations. The average value of ε was estimated from the calibration curve obtained from the absorbance of five solutions with known concentrations, yielding ε = 1.012 × 10^4^ M^–1^ cm^–1^ (*R* ^2^ = 0.9952, Figure S4). The concentration of desorbed dye in the photoanodes was calculated using eq 4 (Figure S5, Supporting Information). These values were subsequently converted into the amount of desorbed dye per unit area of TiO_2_ film, using the relation:

4 $$t=\frac{cv}{s}$$

where *t* is the amount of dye desorbed per unit area of TiO_2_ film, *v* is the volume of the desorption solution, and *s* is the active area of the photoanode film. The increased dye loading for higher Ga-content films may be attributed to the larger available surface area resulting from the progressive decrease in particle size (Table 6). In addition, the FTIR spectra suggest stronger interactions between the dye anchoring groups and the TiO_2_ surface for samples prepared with 2% and 4% nominal Ga contents, which may further favor surface charge injection (Figure 5c). However, the high dye loading observed for the photoanodes obtained with 8% and 16% nominal Ga contents does not translate into improved photocurrent, as observed in Table 6 and supported by Figure 5c. At moderate doping levels, particularly for sample with nominal 4% Ga content, the overall absorption intensity increases, and the band broadens slightly toward longer wavelengths (Figure 5b). This enhancement is consistent with its substantially higher dye loading compared with the undoped photoanode (185.7 ± 2.50 vs 64.6 ± 1.95 nmol cm^–2^), while the FTIR analysis further indicates changes in the interaction of dye anchoring groups with the TiO_2_:Ga surface.^27,40^ The following section discusses the relationship between dye loading and photovoltaic performance.

**Table 6:** Photovoltaic Parameters Derived from the *J*–*V* Curves

DSSCs (Nominal Ga content)	*V*_oc_(V)	*J*_sc_ (mA/cm^2^)	FF (%)	η (%)	Dye loading (nmol/cm^2^)
0%	0.66 ± 0.02	6.81 ± 0.38	57.3 ± 1.12	2.58 ± 0.16	64.6 ± 1.95
1%	0.68 ± 0.02	6.91 ± 0.42	59.5 ± 1.19	2.80 ± 0.18	91.4 ± 2.15
2%	0.69 ± 0.02	7.06 ± 0.49	60.3 ± 1.10	2.94 ± 0.22	123.7 ± 2.21
4%	0.77 ± 0.04	8.45 ± 0.45	63.6 ± 1.33	4.14 ± 0.32	185.7 ± 2.50
8%	0.69 ± 0.01	2.17 ± 0.24	62.1 ± 1.21	0.93 ± 0.10	214.1 ± 2.65
16%	0.69 ± 0.02	1.62 ± 0.18	59.4 ± 1.22	0.66 ± 0.08	149.2 ± 2.33

### 3.1 DSSC Performance

The photovoltaic performance of DSSCs fabricated with undoped TiO_2_ and Ga-doped TiO_2_ photoanodes was systematically evaluated using current density–voltage (*J*–*V*) measurements, as presented in Figure 6a. The extracted photovoltaic parameters were used to assess the influence of incorporated Ga on device efficiency and overall photovoltaic performance. Table 6 summarizes these results, highlighting the performance enhancement achieved through Ga doping relative to the undoped TiO_2_-based DSSC.

**Figure 6:**
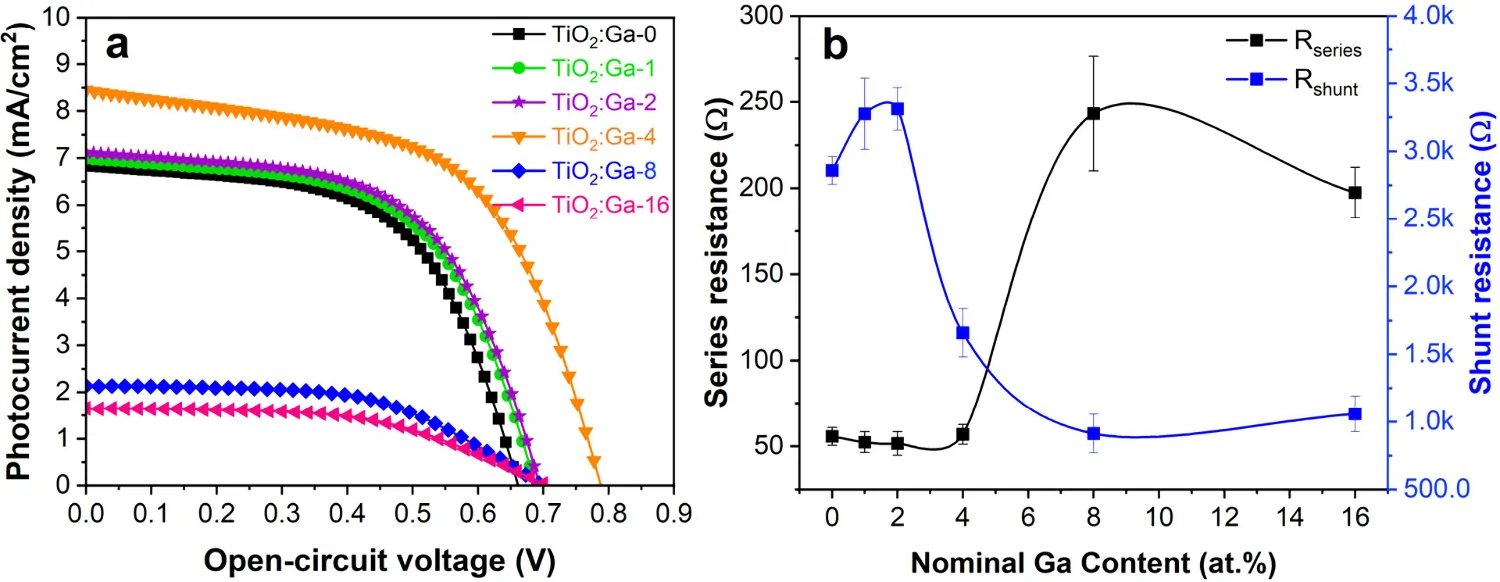
(a) *J*–*V* characteristics plot and (b) series and shunt resistance derived from the *J*–*V* curves of DSSCs incorporating undoped TiO_2_ and Ga-doped TiO_2_ photoanodes.

The device fabricated with undoped TiO_2_ exhibited moderate photovoltaic performance, producing a short-circuit current density (*J*_SC_) of approximately 6.8 mA/cm^2^ and an open-circuit voltage (*V*_OC_) close to 0.66 V. The maximum power point occurred at about 0.40 V, with a corresponding current density of ∼6 mA/cm^2^, resulting in a power conversion efficiency (PCE) of 2.58% and a fill factor (FF) of 57.3%. This moderate efficiency reflects our use of a nonoptimized DSSC structure (no TiCl_4_ post-treatments, additive addition, light-scattering overlayers, or antireflective coatings), but it does not invalidate the relative comparison between doped and undoped devices. In comparison, DSSCs fabricated with Ga-doped TiO_2_ photoanodes with nominal 1% and 2% Ga content showed only marginal PCE improvements relative to the undoped devices, suggesting that the combined effect of improvements in charge injection and light harvesting processes for the Ga incorporation at lower concentrations does not significantly alter the overall PCE. In contrast, DSSCs fabricated with TiO_2_ photoanodes containing nominal 4% Ga showed a substantial improvement in photovoltaic performance, producing a significantly higher *J*_SC_ and *V*_OC_ of 8.45 mA/cm^2^ and 0.77 V, respectively. In addition, the maximum power point shifted toward higher operating values, with *V*_max_ of 0.55 V and a corresponding *J*_max_ of 7.5 mA/cm^2^. Consequently, the overall PCE reached approximately 4.14%, representing nearly a 60% enhancement over the undoped TiO_2_ device. This performance gain was further supported by an enhanced FF of 63.6%, indicating more efficient charge generation under illumination due to optimized Ga content in the nominal 4% Ga sample.

The pronounced improvement observed for the nominal 4% Ga content photoanode is consistent with the resistive parameters extracted from the *J*–*V* characteristics (Figure 6b and Table 7) of the fabricated device. This composition maintains a relatively low series resistance (*R*_s_ = 57.9 Ω), while its shunt resistance (*R*_sh_ = 1658.7 Ω), although lower than that of the nominal 0–2 mol % samples, remains significantly higher than that of the 8% (912 Ω) and 16% (1057.8 Ω) devices. This combination suggests that resistive losses remain limited and leakage pathways are less severe than in the highly doped devices, thereby favoring efficient charge injection and charge collection. Under these conditions, photoinduced charge generation and overall device efficiency improved substantially. In contrast, DSSCs fabricated with higher Ga-content (8% and 16% nominal) TiO_2_ films exhibited a pronounced deterioration in all evaluated photovoltaic parameters, as summarized in Tables 6 and 7. In these devices, *J*_SC_ decreased to 2.17 mA/cm^2^ for the device prepared with a nominal 8% Ga-doped TiO_2_ film and to 1.62 mA/cm^2^ for the device prepared with a nominal 16% Ga-doped TiO_2_ film. Despite this reduction in photocurrent density and overall efficiency, *V*_OC_ increased slightly, reaching ∼0.69 V for both devices. The FF remained pretty similar to that of the undoped device. Consequently, the PCE values decreased to approximately 0.8% and 0.6% for the devices fabricated with nominal 8% and 16% Ga films, respectively. As shown in Figure 6 and Tables 6 and 7, the samples with nominal 8% and 16% Ga exhibit a marked increase in series resistance, approximately 5 times higher than that of the undoped device, together with a substantial reduction in shunt resistance by nearly a factor of 3. This combination reflects increased resistive losses and nonideal current pathways, which hinder efficient charge extraction and promote current leakage. As a result, the strong reduction in *J*_SC_ becomes the dominant factor governing the overall efficiency loss. This behavior is directly correlated with the decrease in FTIR peak intensity of COO^–^ bands observed in Figure 5c, indicating weaker TiO_2_:Ga/dye interfacial coupling. The poorer performance of photoanodes with nominal Ga contents of 8% and 16% is consistent with doping-induced modifications to the electronic landscape of TiO_2_. Higher nominal Ga contents may introduce significant lattice disorder and alter the density and distribution of defect states, potentially including oxygen vacancies and Ti^3+^-related trap states. These defect states can adversely affect electron injection efficiency and promote interfacial recombination with the electrolyte, thereby reducing *J*_SC_ and overall PCE despite the moderate increase in *V*_OC_.^28,30,46^

**Table 7:** Series and Shunt Resistances Estimated from the *J*–*V* Characteristics

DSSCs (Nominal Ga content)	Series resistance (Ω)	Shunt resistance (Ω)
0%	55.7 ± 5.2	2858.1 ± 101.9
1%	52.4 ± 6.1	3275.4 ± 261.8
2%	53.6 ± 6.9	3331.7 ± 156.6
4%	57.9 ± 5.8	1658.7 ± 178.2
8%	243.2 ± 33.2	912.4 ± 142.8
16%	197.4 ± 14.6	1057.8 ± 133.2

In contrast, the enhanced photovoltaic response at moderate Ga concentrations, particularly in samples with nominal 4% Ga content, can be attributed to the combined effect of improved dye loading and enhanced electron injection efficiency promoted by Ga-induced microstructural modifications, as discussed in the material characterization section. The progressive reduction in average particle size and the increase in specific surface area provide more active adsorption sites for dye anchoring, thereby enhancing light harvesting and facilitating interfacial charge-transfer processes at the TiO_2_:Ga/dye interface. However, the high dye loading observed in the sample with nominal 8% Ga (Table 6) does not translate into improved photovoltaic performance. Despite adsorbing more dye, these devices show markedly lower *J*_SC_, *V*_OC_, and PEC, indicating that charge-injection limitations and recombination losses could dominate at higher nominal Ga contents. This interpretation is consistent with the increase in series resistance, the decrease in shunt resistance, inefficient dye–semiconductor coupling, and the greater structural disorder observed at high nominal Ga contents. Therefore, the optimum performance at nominal 4% Ga arises from a balance between optimum dye adsorption and favorable charge-injection properties, rather than from dye loading alone. Overall, these results demonstrate that the photovoltaic response at high nominal Ga contents is governed by the competition between beneficial surface-related effects and detrimental electronic limitations.^47–50^

The photovoltaic response of Ga-doped TiO_2_-based DSSCs therefore clearly depends on dopant concentration, revealing an optimal doping regime at intermediate nominal Ga contents. While low Ga contents (e.g., 1–2% nominal) produced only marginal improvements relative to undoped TiO_2_, excessive Ga incorporation (e.g., 8% and 16% nominal) led to severe deterioration of device performance, mainly due to substantial losses in photocurrent density despite a moderate increase in *V*_OC_.^30,47^ These observations indicate that Ga content strongly influences the balance among light harvesting, charge injection, and charge collection.

Figure 7 shows the stability evolution of the photovoltaic parameters of the fabricated DSSCs over 30 days. All devices were tested under identical conditions, and the reported values represent the average of 5 cells per batch. All devices showed a modest increase in all PV parameters during the first few days, reaching maximum efficiency around day 4, followed by a gradual decrease or stabilization, depending on the nominal Ga content. Similar initial increases in DSSC photovoltaic performance after light soaking or temperature stress have been reported for DSSCs employing different dyes, electrolytes, and photoanode materials.^51–55^ Listori et al. attributed the increase in *J*_SC_ after light soaking to a downward shift of the TiO_2_ conduction band, which can enhance the electron-injection rate, although a simultaneous decrease in *V*_OC_ was observed.^51^ Wang et al. associated the increase in FF after temperature stress with activation or improved contact between the electrolyte and the Pt counter electrode.^52^ Cabau et al. related the increase in *V*_OC_ to increased electron lifetime resulting from improved TiO_2_/dye interaction after light soaking.^53^ In the present study, we did not perform independent measurements to directly verify electrolyte infiltration or dye reorganization during the initial aging period. Therefore, gradual electrolyte infiltration into the mesoporous TiO_2_ network, rearrangement or partial desorption of dye molecules, and progressive equilibration of the TiO_2_/dye/electrolyte interfaces may have contributed to the initial increase in photovoltaic performance.^54,55^

**Figure 7:**
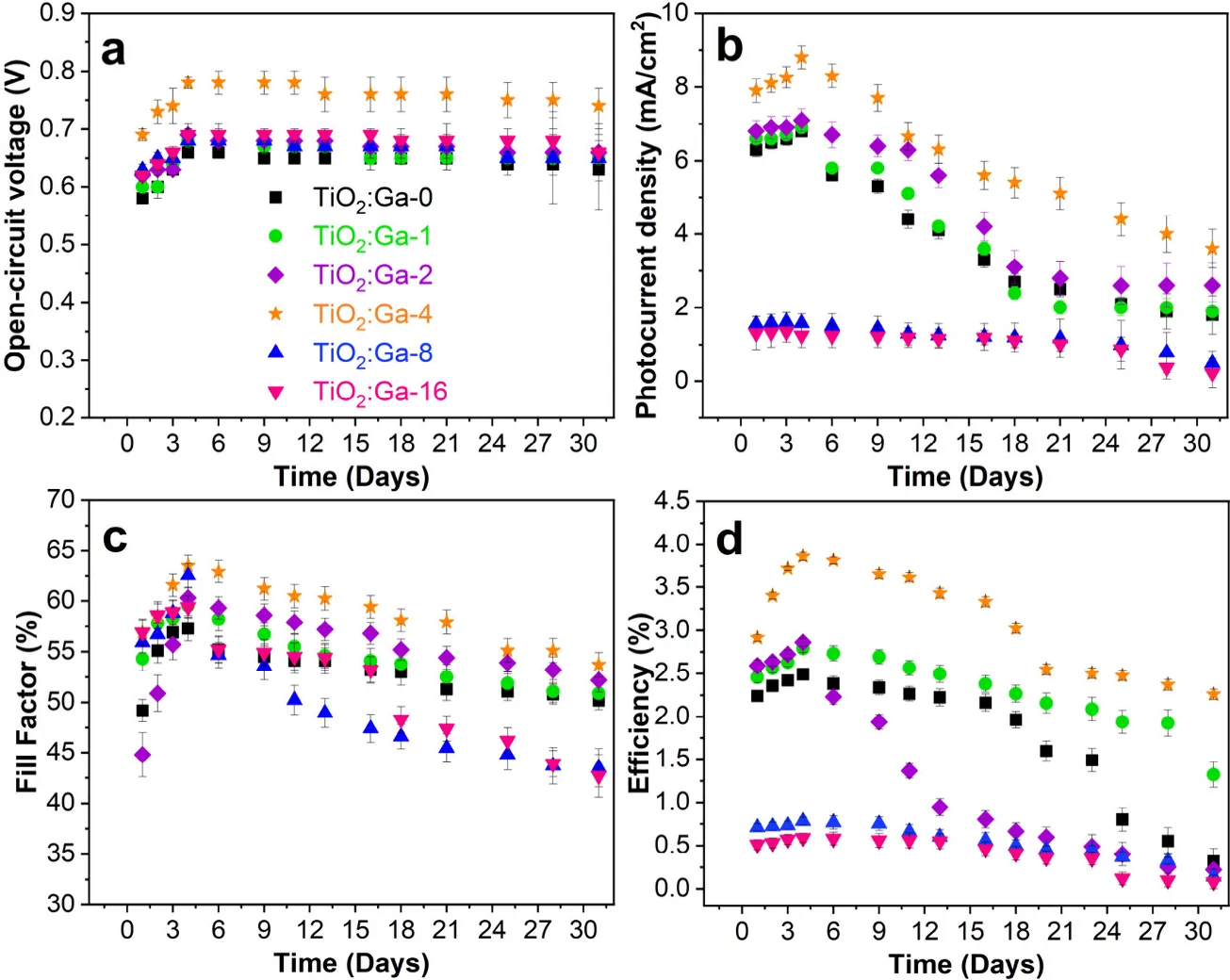
Evolution of photovoltaic parameters for DSSCs with undoped and Ga-doped TiO_2_ photoanodes: (a) open-circuit voltage, (b) short-circuit current density, (c) fill factor, and (d) power conversion efficiency.

Among the evaluated parameters, *V*_OC_ remained the most stable, displaying only minor fluctuations throughout the testing period, as shown in Figure 7a. Since *V*_OC_ is governed by the energy difference between the TiO_2_ quasi-Fermi level and the redox potential of the electrolyte, this behavior indicates that the interfacial energetics and recombination kinetics under open-circuit conditions were largely preserved. The performance degradation was primarily driven by the evolution of *J*_SC_, which began to decrease after approximately the fourth day, particularly for the undoped device and those with low nominal Ga contents (1–4% nominal), as shown in Figure 7b. Such photocurrent losses are typically associated with partial dye desorption and gradual modification of the TiO_2_/dye interface, both of which reduce light-harvesting and electron-injection efficiencies. Surface modifications may also introduce additional recombination pathways that limit charge collection.^48,50,56,57^ Devices with higher nominal Ga contents (8% and 16%) exhibited comparatively higher stability in *J*_SC_, although their initial photocurrent values were lower than those of the low-Ga-doped devices. As can be noticed in Figure 7b, after 30 days of testing, the *J*_SC_ values of these highly doped devices decreased by approximately 50%, leading to even lower overall efficiency (Figure 7d).

As shown in Figure 7c, the fill factor decreased steadily for all compositions after day 4, consistent with higher resistive losses and deviations from ideal charge-transport behavior during device aging. This behavior is commonly associated with electrolyte diffusion limitations, enhanced interfacial recombination, and possible deterioration of the catalytic activity of the Pt counter electrode.^46,47^ The evolution of efficiency followed the same trend as *J*_SC_ (Figure 7d), confirming that photocurrent losses dominated long-term degradation of device performance, while voltage-related parameters remained comparatively unaffected. The photovoltaic parameters obtained after 30 days of testing further support this observation, revealing a substantial reduction in photocurrent with only minor changes in *V*_OC_. Finally, as observed in Figure 7, the device based on TiO_2_ with 4% nominal Ga maintained the best photovoltaic performance after 30 days of testing, exhibiting slightly improved long-term stability compared with the other evaluated devices.^34,35,46–48^

To gain further insight into recombination processes, open-circuit voltage decay (OCVD) measurements were performed on DSSCs fabricated with undoped and Ga-doped TiO_2_ photoanodes. This technique measures the temporal decay of the photovoltage after illumination is switched off under open-circuit conditions, providing information on electron lifetime and recombination kinetics.^58,59^

In the OCVD, the electron lifetime, τ_e_, is related to the voltage decay, *V*_OC_(*t*), by the expression:^59^

5 $$\tau_{e}=-\frac{k_{B}T}{\alpha e}{\left(\frac{dV_{\mathrm{OC}}}{dt}\right)}^{-1}$$

where *k*_B_ *T* represents the thermal energy, *e* denotes the elementary positive charge, and α is a parameter related to the average depth of the trap-state distribution below the conduction band.

By analyzing τ_e_ as a function of the *V*_OC_, it is possible to evaluate how Ga incorporation into the TiO_2_ structure influences charge-recombination dynamics within the photoanode and, consequently, overall DSSC performance. The trap-distribution exponent, α, is determined from the electron lifetime versus voltage plot, where the slope in the semilogarithmic representation is directly related to α.^59^ Consequently, any variation in the slope reflects changes in the distribution of trap states below the conduction band of Ga-doped TiO_2_.

Figure 8a presents the electron lifetime as a function of voltage for the undoped TiO_2_ photoanode and photoanodes with 4% and 16% nominal Ga contents. For all samples, τ_e_ decreases systematically with increasing voltage, a behavior commonly associated with accelerated recombination at higher electron densities in the TiO_2_ semiconductor. This trend reflects the increased probability of electron transfer to oxidized species in the electrolyte as the quasi-Fermi level shifts upward due to the electron trap-energy distribution below the TiO_2_ conduction band.^30,58,59^ Among the studied compositions, the photoanodes with 4% nominal Ga content exhibited consistently longer electron lifetimes at voltages above 0.5 V, as observed in Figure 8a. This behavior indicates more effective suppression of recombination processes, consistent with the higher *V*_OC_ values reported in Table 6 and Figure 7a. The extended τ_e_ suggests that moderate Ga incorporation improves the electronic quality of the photoanode, likely by reducing trap-assisted recombination pathways or modifying the interfacial energetics.^8,60^

**Figure 8:**
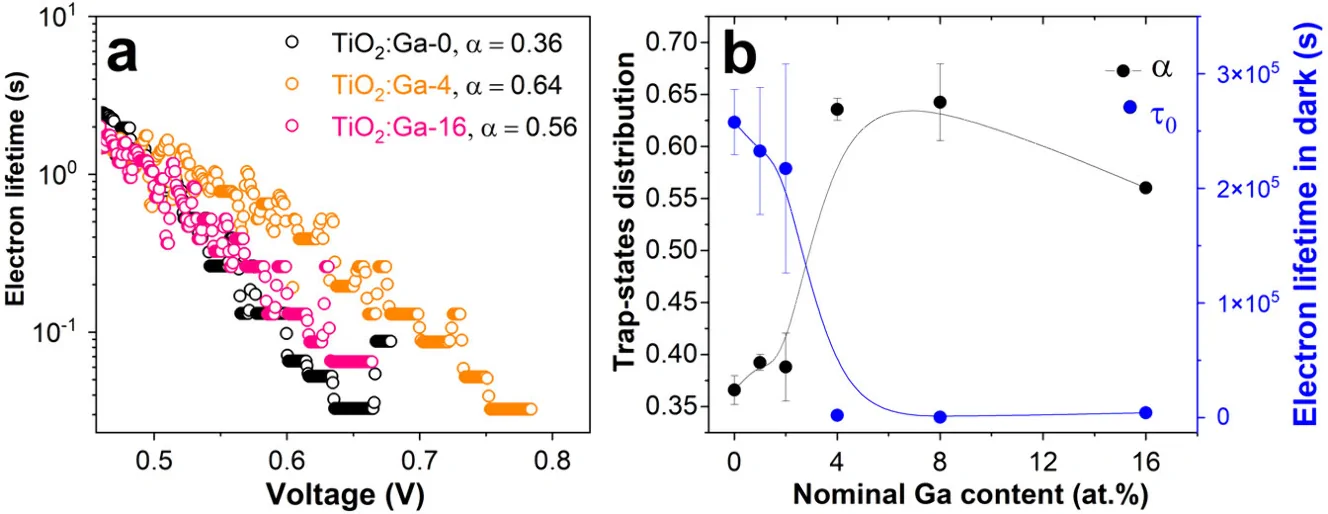
(a) Electron lifetime as a function of voltage, and (b) trap-state distribution parameter and electron lifetime in the dark as a function of nominal Ga content.

Figure 8b summarizes the OCVD analysis, from which the electron lifetime in the dark (τ_0_) and the trap-distribution parameter α were extracted according to reference.^59^ Both parameters are closely related to recombination kinetics, and their evolution provides insight into how Ga doping affects charge-recombination dynamics. Samples with nominal Ga contents of 0, 1, and 2% exhibited τ_0_ values comparable to those of the undoped device, indicating that low doping levels do not significantly alter the dark recombination lifetimes. In contrast, higher nominal Ga contents (8% and 16% nominal) resulted in a clear reduction in τ_0_ (Figure 8b), accompanied by lower photovoltage values (Table 6) than those observed for the photoanode with nominal 4% Ga content. This behavior suggests that excessive Ga incorporation promotes the formation of additional recombination centers, possibly associated with lattice distortion, defect states, or dopant segregation, which outweigh the beneficial effects observed at intermediate doping levels.

On the other hand, the results shown in Figure 8b indicate that the α parameter gradually increases from approximately 0.36 for the undoped TiO_2_ devices to 0.64 for the devices with 8% nominal Ga content and then decreases to 0.56 for the devices with 16% nominal Ga content, consistent with the fitted values reported in Figure 8a. This behavior suggests the formation of a deeper trap-state distribution below the TiO_2_ conduction band with increasing Ga incorporation. Simultaneously, the electron lifetime in the dark decreases by approximately 3 orders of magnitude as the Ga concentration increases, indicating a significant increase in the recombination rate under dark conditions. However, the redistribution of trap states toward deeper energy levels partially compensates for the reduced dark electron lifetime. As a result, at voltages close to the maximum power point (e.g., voltages higher than 0.5 V), the electron lifetime for the TiO_2_ electrode with 4% nominal Ga content becomes longer than that of the undoped electrode and electrodes with nominal 16% Ga content, as observed in Figure 8a. This behavior also helps to explain why devices containing high Ga contents (e.g., 8% and 16% nominal) maintain higher open-circuit voltages than the undoped devices (Table 6). The results presented indicate that an optimal nominal Ga content of approximately 4% produces a favorable redistribution of trap states toward deeper energy levels, reducing recombination losses under DSSC operating voltages. As the results indicate, higher specific surface area or higher dye adsorption are not the only criteria for achieving better DSSC performance. Beyond dye loading and specific surface area, DSSC performance depends strongly on charge injection efficiency and the electronic-state distribution in the photoanode band gap, which regulate carrier recombination and carrier lifetime.

## 4 Conclusions

Ga-doped TiO_2_ nanoparticles with different nominal Ga contents (0–16%) were successfully synthesized using a rapid microwave-assisted method. Structural and optical analyses revealed systematic Ga-induced modifications of the anatase TiO_2_ structure, leading to reduced crystallite and particle sizes, and a significant increase in specific surface area. Moderate nominal Ga content also enhanced dye loading and visible-light absorption without significantly altering the TiO_2_ bandgap. Among all compositions, the TiO_2_ photoanode with 4% nominal Ga content exhibited the best photovoltaic performance and stability in DSSCs, achieving improved photocurrent, open-circuit voltage, and overall efficiency. These improvements are attributed to a better coupling between enhanced light harvesting, increased charge injection, and favorable modification of trap-state distribution, which reduced charge recombination under operating conditions. The results demonstrate that controlled Ga doping is an effective strategy for optimizing the structural and electronic properties of TiO_2_ photoanodes. However, higher Ga contents were associated with the formation of a deeper trap-state distribution and inefficient dye–semiconductor coupling that deteriorate device performance. The synthesis approach and porous nanostructures developed in this work may also be promising for other applications such as photocatalysis, lithium-ion batteries, and catalyst–support materials.

## Supplementary Material



## Notes

Reactor temperature profile curve during the microwave-assisted synthesis; variation of lattice parameter c and crystallite size as a function of nominal Ga content; BJH pore size distribution plots for the undoped and Ga-doped TiO_2_ nanoparticles obtained from their N_2_ adsorption curves; calibration curve of Z907 absorbance as a function of Z907 dye concentration; calibration curve of Z907 absorption in Ga-doped TiO_2_ film as a function of dye concentration (5–25 μM) prepared from UV–vis absorption spectra of dye-desorbed solutions (PDF)
