# Supplementary material for: Effect of Ga Doping on Microwave-Assisted Synthesized TiO2 Nanoparticles and their Photovoltaic Performance as DSSC Photoanodes
Source: ACS Omega. 2026 Oct 27;11(44):68551–64. doi: 10.1021/acsomega.6c06567 (PMC13756734; doi:10.1021/acsomega.6c06567)
Supplement: Supplementary file 1 [file ao6c06567_si_001.pdf]

Supporting Information

Effect of Ga doping on Microwave-Assisted Synthesized TiO_2 Nanoparticles and their Photovoltaic Performance as DSSC Photoanodes

J. Arriola-Oliva,[1] E. Regalado-Pérez,[2] U. Pal,[2,*] J. Villanueva-Cab[2,*]

[1] Instituto de Ciencias Químicas, Benemérita Universidad Autónoma de Puebla, San Pedro Zacachimalpa 72960 Puebla, México.

[2] Instituto de Física "Ing. Luis Rivera Terrazas", Universidad Autónoma de Puebla, 18 Sur y Av. San Claudio, C.U., 72570 Puebla, México.

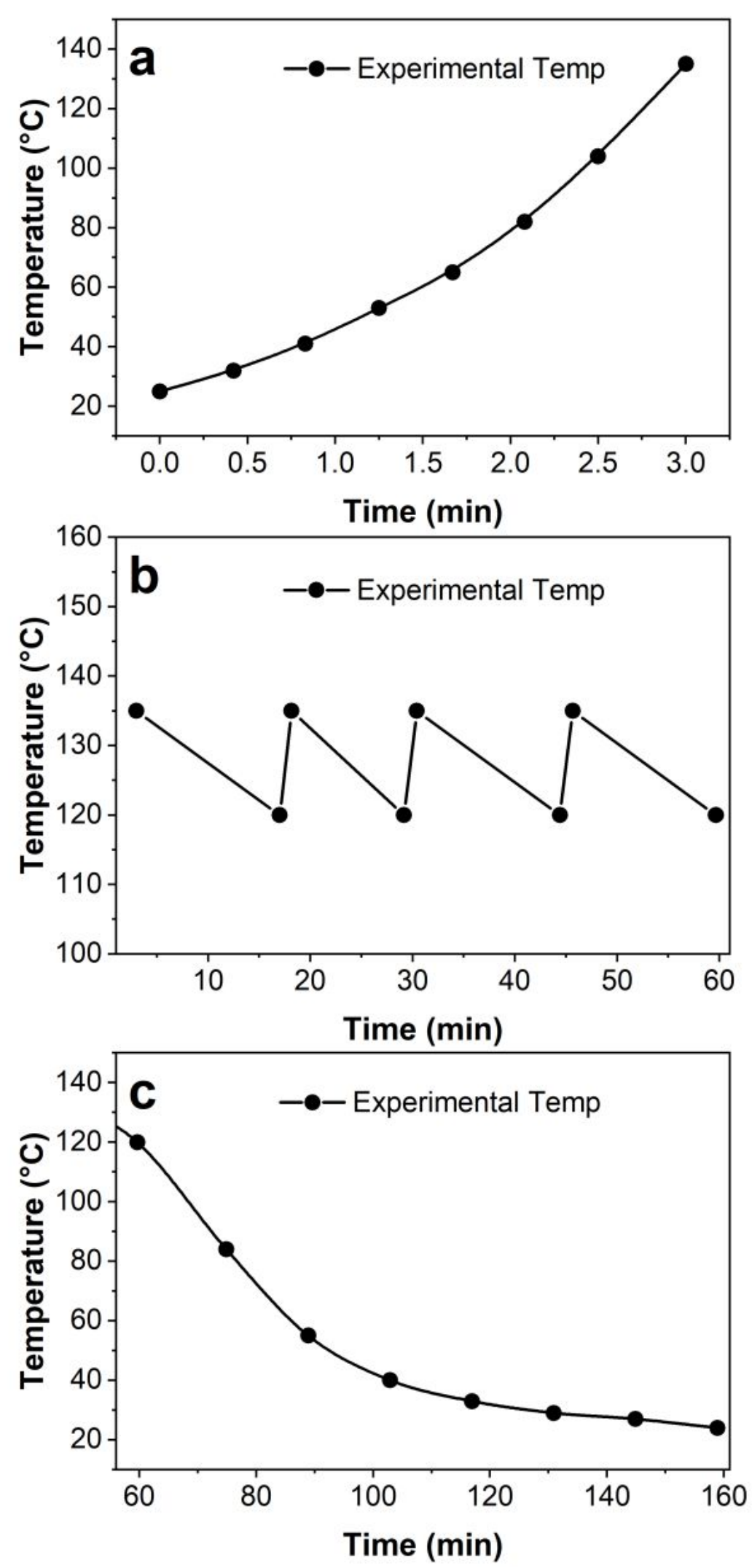

Figure S1. Reactor temperature profile curve during the microwave-assisted synthesis. a) Heating ramp: Initial heating from room temperature to 135 °C in 3 minutes, pulsed radiation cycles (8s ON/25 s OFF). b) Heating/Cooling: The temperature

was maintained in the target range (120–135 °C) using pulsed radiation cycles (8s ON/25 s OFF) over the reaction hold time. c) Cooling stage: Natural cooling profile back to room temperature after switching off the microwave system.

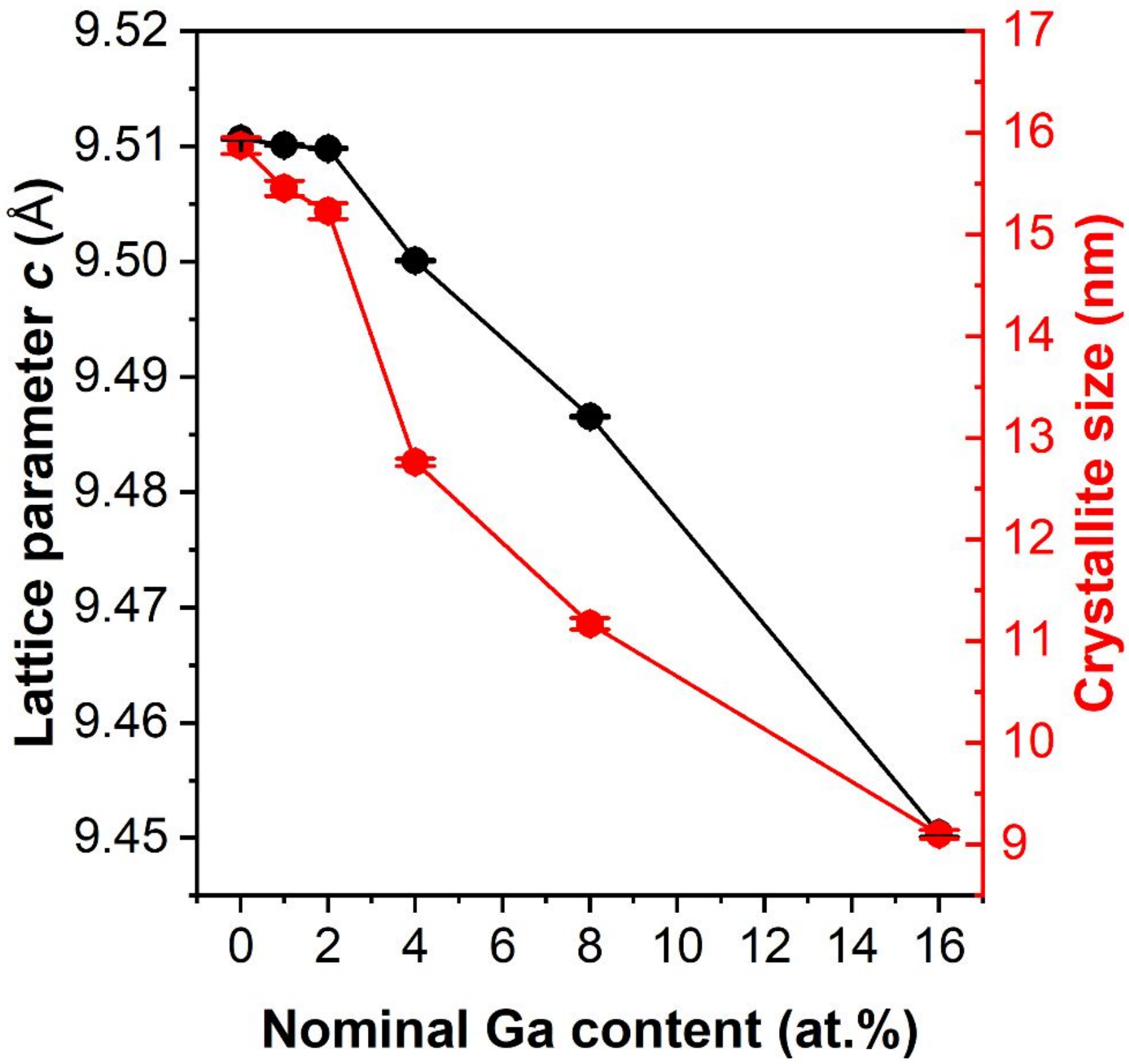

Figure S2. Variation of lattice parameter c and crystallite size as a function of Ga doping concentration.

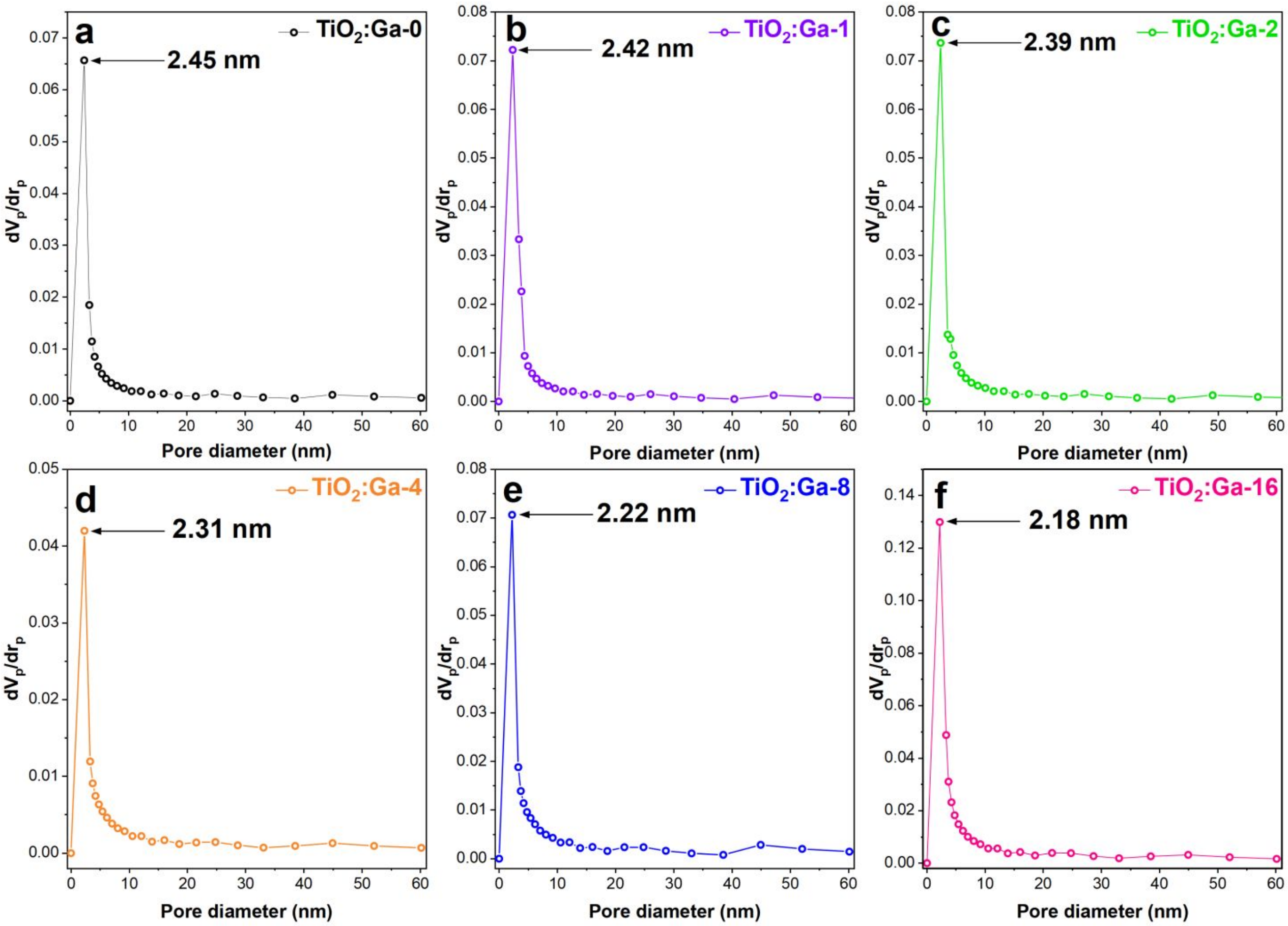

Figure S3. BJH pore size distribution plots obtained from the N_2 adsorption curves for the nanoparticles prepared with a) 0%, b) 1%, c) 2%, d) 4%, e) 8%, and f) 16% nominal Ga content. Annotated values denote the peak modal pore diameter for each sample.

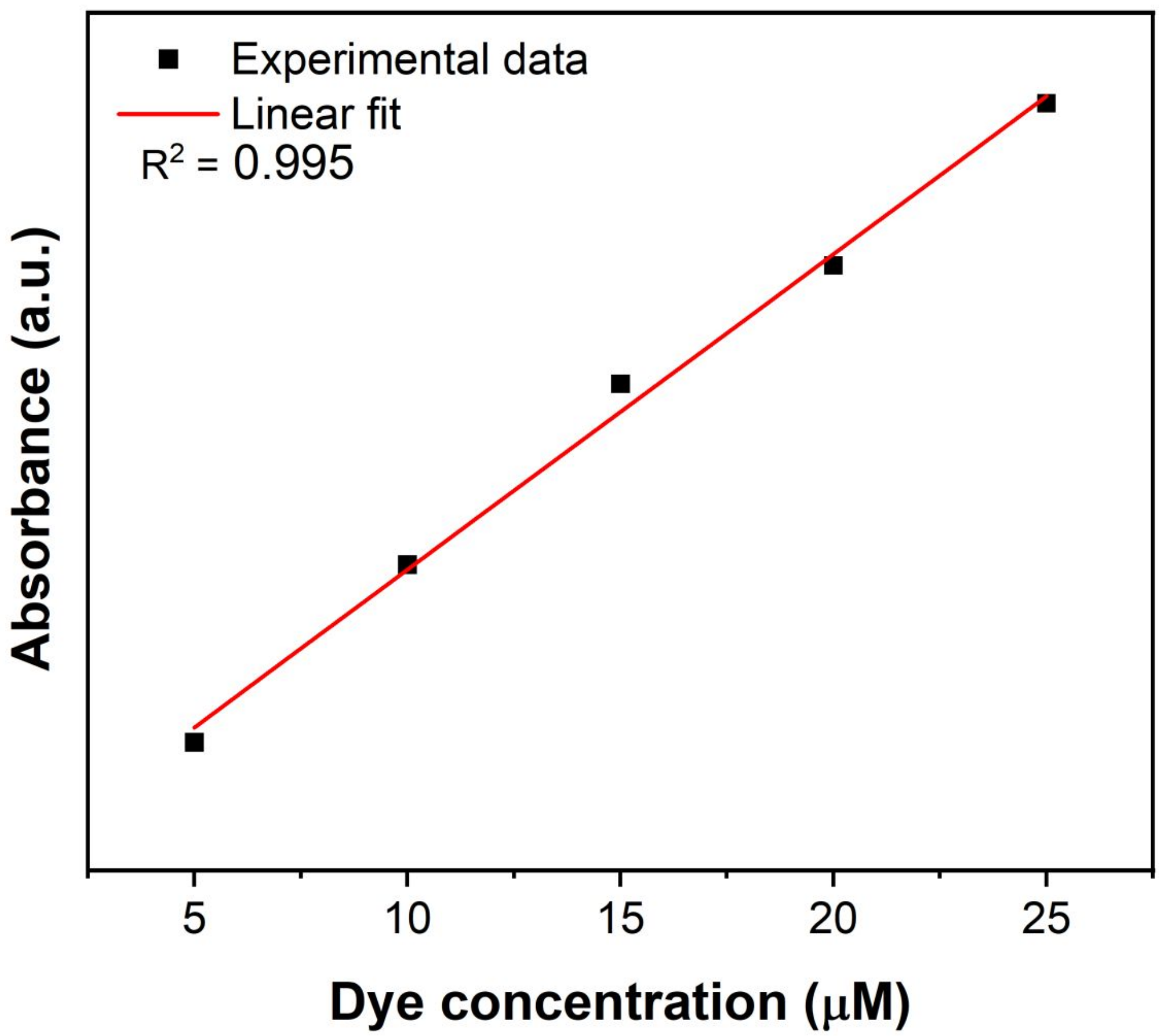

Figure S4. Calibration curve of Z907 absorbance as a function of Z907 dye concentration (5-25 μM). The linear fit according to the Lambert-Beer Law (R^2 = 0.995)

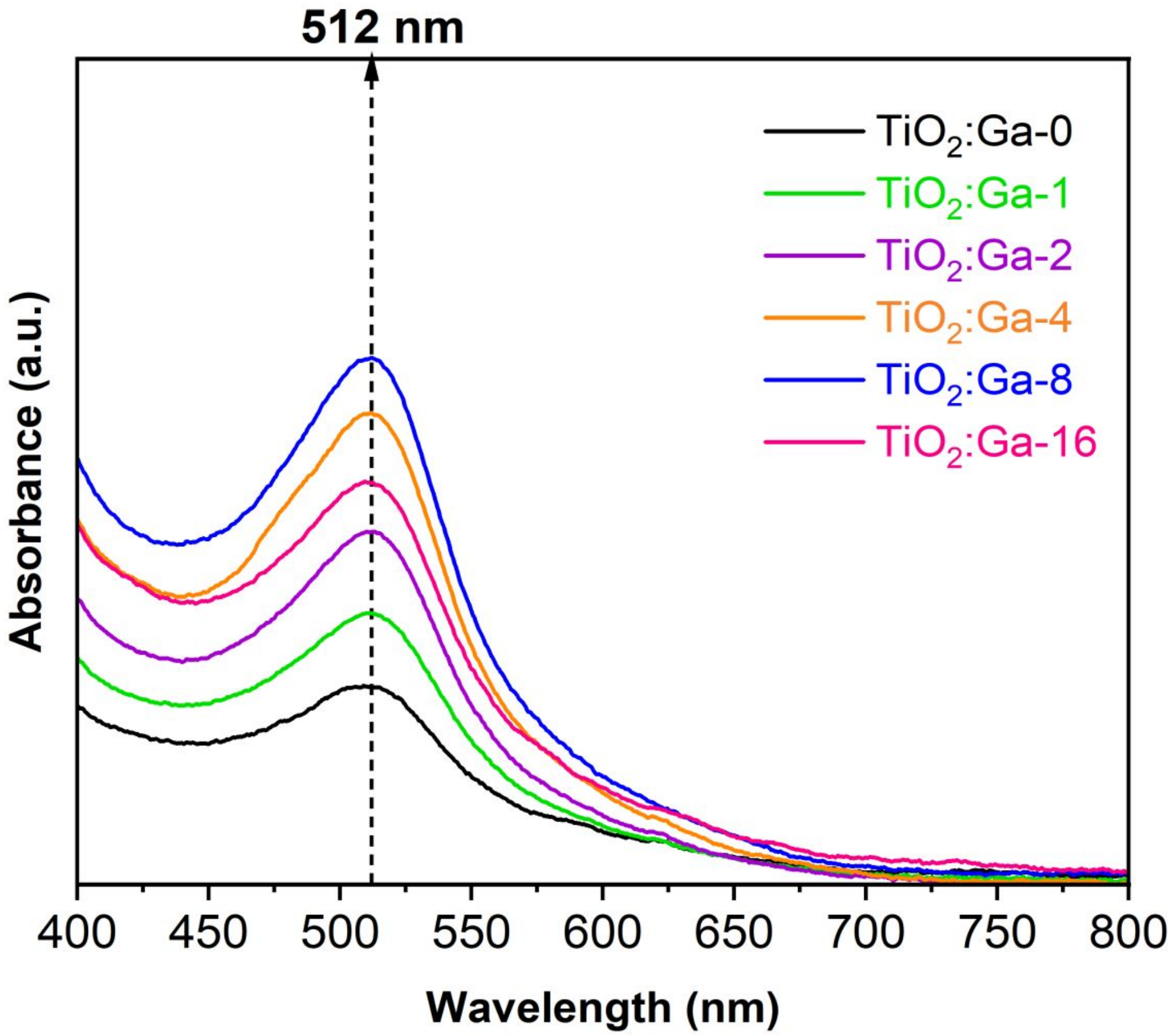

Figure S5. UV-Vis absorption spectra of dye-desorbed solution for undoped TiO_2 and Ga-doped TiO_2 photoanodes with varying nominal Ga content (1- 16%). The vertical dashed line highlights the characteristic absorption maximum of the dye at 512 nm. The maximum absorbance increases up to 8% nominal Ga content due to enhanced dye loading capacity, followed by a slight decrease at 16% nominal Ga content.
